# METTL3-mediated N^6^-methyladenosine modification of STAT5A promotes gastric cancer progression by regulating KLF4

**DOI:** 10.1038/s41388-024-03085-2

**Published:** 2024-06-15

**Authors:** Yichen Zang, Zhuangfei Tian, Dandan Wang, Yaxuan Li, Wenhui Zhang, Cunying Ma, Zhenzhi Liao, Wenrong Gao, Lilin Qian, Xia Xu, Jihui Jia, Zhifang Liu

**Affiliations:** 1 Department of Biochemistry and Molecular Biology, Key Laboratory for Experimental Teratology of Chinese Ministry of Education, School of Basic Medical Sciences, Cheeloo College of Medicine, Shandong University, Jinan, China; 2 Department of Microbiology, Key Laboratory for Experimental Teratology of Chinese Ministry of Education, School of Basic Medical Sciences, Cheeloo College of Medicine, Shandong University, Jinan, China; 3 School of Clinical Medicine, Qingdao University, Qingdao, China

**Keywords:** Gastric cancer, Epigenetics

## Abstract

N^6^-methyladenosine (m^6^A) is the predominant post-transcriptional RNA modification in eukaryotes and plays a pivotal regulatory role in various aspects of RNA fate determination, such as mRNA stability, alternative splicing, and translation. Dysregulation of the critical m^6^A methyltransferase METTL3 is implicated in tumorigenesis and development. Here, this work showed that METTL3 is upregulated in gastric cancer tissues and is associated with poor prognosis. METTL3 methylates the A2318 site within the coding sequence (CDS) region of STAT5A. IGF2BP2 recognizes and binds METTL3-mediated m^6^A modification of STAT5A through its GXXG motif in the KH3 and KH4 domains, leading to increased stability of STAT5A mRNA. In addition, both METTL3 and IGF2BP2 are positively correlated with STAT5A in human gastric cancer tissue samples. *Helicobacter pylori* infection increased the expression level of METTL3 in gastric cancer cells, thereby leading to the upregulation of STAT5A. Functional studies indicated that STAT5A overexpression markedly enhances the proliferation and migration of GC cells, whereas STAT5A knockdown has inhibitory effects. Further nude mouse experiments showed that STAT5A knockdown effectively inhibits the growth and metastasis of gastric cancer in vivo. Moreover, as a transcription factor, STAT5A represses KLF4 transcription by binding to its promoter region. The overexpression of KLF4 can counteract the oncogenic impact of STAT5A. Overall, this study highlights the crucial role of m^6^A in gastric cancer and provides potential therapeutic targets for gastric cancer.

## Introduction

Among all types of cancers, gastric cancer (GC) is ranked fifth in terms of global incidence and fourth in terms of mortality [1]. The burden of this disease is predicted to increase by 62% by 2040 [2]. Patients from East and Southeast Asia accounted for approximately two-thirds of GC patients diagnosed in 2020 [1]. Persistent infection with *Helicobacter pylori (H. pylori)* and the subsequent development of severe gastritis and peptic ulcers account for a substantial proportion of gastric adenocarcinoma cases [3, 4]. Despite advancements in chemotherapy and surgery, the majority of patients diagnosed at advanced stages have poor prognoses due to the absence of clear clinical biochemistry indications [5].

Chemotherapy drugs with reduced toxicity, in combination with molecular targeted therapies, have demonstrated promising potential for optimizing the overall survival (OS) of patients diagnosed with GC [6]. These biomarkers and molecular classifications tailored to GC patients pave the way for more precise diagnoses and treatments [7]. However, the deficiency of more suitable targets is still a disadvantage in clinical drug development [8]. This highlights the importance of investigating the molecular mechanisms related to GC [8, 9].

N^6^-methyladenosine (m^6^A) is the most prevalent, abundant, and evolutionarily conserved post-transcriptional modification primarily observed in eukaryotic messenger RNA (mRNA) [10]. Accumulating evidence has demonstrated that the m^6^A methylation of RNA influences RNA metabolism and actively participates in the pathogenesis of numerous diseases, including cancer progression [11–14]. m^6^A modification is catalyzed by m^6^A methyltransferases (writers), recognized by m^6^A binding proteins (readers), and reversed by demethylases (erasers) [15]. m^6^A modifications to mRNAs or other RNA polymerase II transcripts are introduced by the m^6^A writer complex, where methyltransferase-like 3 (METTL3) serves as the catalytic component and methyltransferase-like 14 (METTL14) functions as a modulator that enhances the enzymatic activity of METTL3 [15–18]. In addition, the m^6^A writer complex also comprises WTAP (the principal METTL3 adaptor), RBM15 (possessing an RNA-binding domain), CBLL1 (to guide mRNA region-selective methylation) and the WTAP interaction partner KIAA1429 [19–21]. The RNA reader protein recognizes m^6^A modifications, interacts with the RNA molecules with m^6^A modification and executes specific biological functions [22]. However, distinct readers exert diverse downstream biological effects [23]. Insulin-like growth factor-2 mRNA-binding proteins (IGF2BPs), including IGF2BP1/2/3, specifically recognize the consensus GG (m^6^A) C sequence [24] and regulate gene expression by stabilizing their target mRNAs in a m^6^A-dependent manner [24, 25]. The KH3 and KH4 homology domains of IGF2BPs play a pivotal role in recognizing m^6^A modifications and facilitating their functions [26, 27]. Emerging evidence shows that dysregulation of m^6^A abundance is frequently observed in diverse cancer types and plays a critical role in cancer initiation, progression, metastasis, drug resistance, and recurrence [28]. It has been reported that global m^6^A mRNA levels are obviously greater in GC tissues than in normal tissues [29]. However, the mechanisms involved in the aberrant expression of these m^6^A-related molecules in GC still need to be elucidated.

In this study, we demonstrated that METTL3 promotes the m^6^A modification of STAT5A and increases its expression in GC cells. Infection with *H.pylori* increases the expression of METTL3 in GC cells, thereby leading to the upregulation of STAT5A. The reader protein IGF2BP2 can recognize and bind to STAT5A with m^6^A modification. Furthermore, our findings indicate that KLF4 is a downstream effector molecule of STAT5A. KLF4 functions as a tumor suppressor and its transcriptional activity is repressed by the binding of STAT5A to its promoter. These findings shed light on the epigenetic regulation of STAT5A mRNA mediated by METTL3 in gastric carcinoma and provided potential target for effective treatment of GC.

## Results

### METTL3 is highly expressed in patients with gastric carcinoma, and *Helicobacter pylori* infection contributes to its upregulation

To evaluate the potential involvement of the m^6^A methyltransferase complex in GC, we initially examined the expression of different members of the m^6^A writer complex using the dataset from The Cancer Genome Atlas (TCGA). Our analysis revealed that METTL3, RBM15, KIAA1429, CBLL1, and WTAP were significantly upregulated in GC tissues compared to the noncancerous tissues (Fig. S1A). We further analyzed their correlation with the overall survival of patients with GC using the data from the Kaplan–Meier Plotter Database and found that METTL3 was most significantly associated with poor prognosis among these upregulated genes (Fig. S1B). We further investigated the expression of METTL3 in the Gene Expression Omnibus (GEO) database and found that METTL3 was also upregulated in GC tissues in the GSE54129 cohort (Fig. S2A). Subsequently, we detected the protein level of METTL3 in 35 pairs of GC and adjacent nontumor tissues, followed by quantification using ImageJ software. Compared with that in nontumor tissues, METTL3 protein expression was significantly upregulated in 74.3% of the GC tissues (Fig. S2B, C). Given the significant association between *H. pylori* infection and GC, we infected GC cells with *H. pylori* to explore whether *H. pylori* infection contributes to the upregulation of METTL3 in GC cells. Our results showed that *H. pylori* infection increased the expression level of METTL3 in a time-dependent manner but had no significant effect on the expression of the demethylases FTO and ALKBH5 (Fig. S2D).

### METTL3 promotes GC cell proliferation and migration through its RNA-binding ability

Next, we investigated the biological role of METTL3 in GC. We used CCK-8 and EdU experiments to detect cell proliferation ability and Transwell assay to test cell migration ability. Our results showed that overexpression of METTL3 promoted the proliferation and migration of GC cells (Fig. S3A–G). In contrast, upon METTL3 knockdown with small interfering RNA (siRNA), the proliferative and migratory capacities of GC cells were significantly decreased (Fig. S3H–N), which is consistent with previous reports [29, 30].

Then, we constructed a mutated vector of METTL3 with an impaired RNA binding domain [18] to investigate its relevance to the oncogenic function of METTL3 in GC cells. Our results revealed that the ability of WT-METTL3 to promote cell proliferation and migration was significantly attenuated by the METTL3 mutation (Fig. S4A–E). These findings indicated that the tumor-promoting effect of METTL3 in GC depended on its RNA- binding ability.

### STAT5A acts as a downstream target of METTL3

**Fig. 1:**
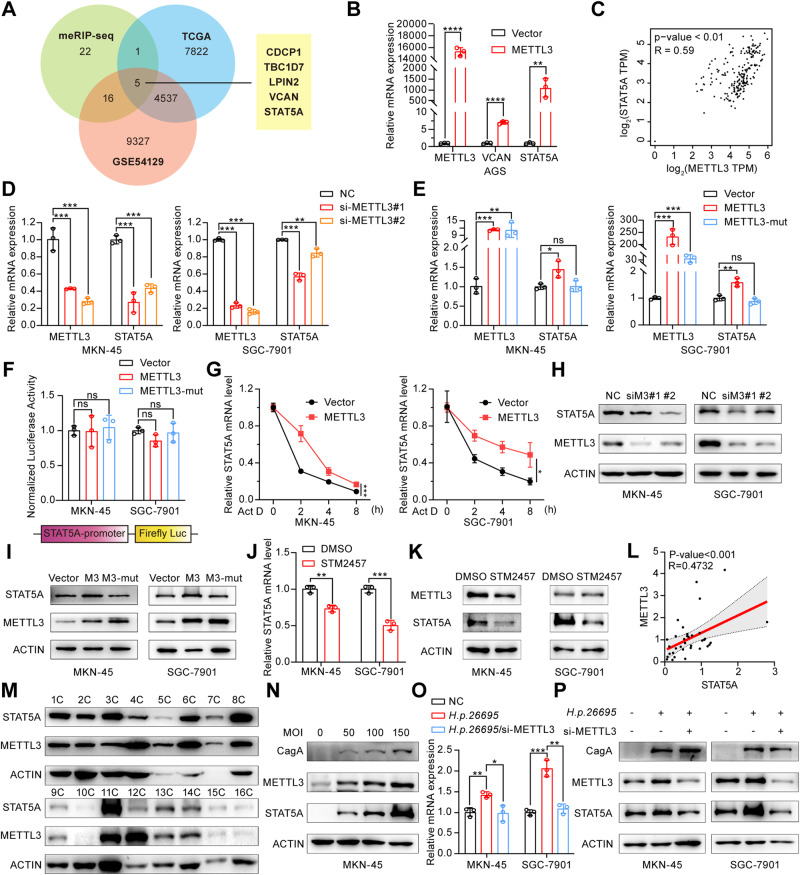
METTL3 enhances the expression of STAT5A. **A** Venn diagram illustrating the intersection of upregulated genes in GC tissues based on data from the TCGA and GEO (GSE54129) databases and the genes with reduced m^6^A levels detected by MeRIP-Seq upon METTL3 knockdown. The inclusion criterion for the data from TCGA and GEO databases (GSE54129) is that the gene expression levels in GC tissues are more than 2 times that in normal tissues (FC > 2, *P* < 0.01). The inclusion criterion for MeRIP-Seq is a 150-fold decrease in m^6^A levels upon METTL3 knockdown. **B** The relative mRNA expression of the candidate genes screened in (A) was assessed by RT-qPCR in AGS cells overexpressing METTL3. **C** Correlation between METTL3 and STAT5A expression in normal gastric tissues from the GEPIA (http://gepia.cancer-pku.cn/) platform was analyzed using Spearman’s test. **D** RT-qPCR was used to determine the relative mRNA expression of STAT5A and METTL3 in MKN-45 and SGC-7901 cells transfected with negative control siRNA (NC) or METTL3 siRNA (si-METTL3). **E** qRT-PCR was used to determine the relative mRNA expression of METTL3 and STAT5A in MKN-45 and SGC-7901 cells transfected with empty vector (Vector), wild-type (METTL3) or mutated METTL3 (METTL3-Mut) expression vectors. **F** Detection of relative dual-luciferase activity in GC cells cotransfected with the pRL-TK and the pGL3-STAT5A promoter luciferase reporter vector, together with the wild-type or mutant METTL3 expression vector. **G** STAT5A mRNA stability was determined in MKN-45 and SGC-7901 cells transfected with empty vector or METTL3 expression vector prior to the addition of Act D for 0, 2, 4, or 8 h. **H** Western blot analysis of STAT5A expression in GC cells transfected with METTL3 siRNA. NC: negative control siRNA; siM3: METTL3 siRNA. **I** Western blot analysis of the expression of STAT5A in GC cells transfected with the wild-type or mutant METTL3 expression vector. M3: WT-METTL3 expression vector; M3-Mut: mutant METTL3 expression vector. **J** RT-qPCR analysis of STAT5A and METTL3 mRNA in MKN-45 and SGC-7901 cells treated with 10 μM STM2457 for 72 h. **K** Western blot analysis of STAT5A and METTL3 protein in GC cells treated with 10 μM STM2457 for 72 h. **L** Correlation analysis of METTL3 and STAT5A protein expression in GC tissues from 35 patients. The data comes from the ratio of the Western blot band density of STAT5A and METTL3 to the corresponding β-actin. **M** Representative Western blot results of STAT5A and METTL3 expression in GC tissues. **N** Western blot was used to detect the expression of STAT5A and METTL3 in MKN-45 cells infected with *H.pylori 26695* at a MOI of 0, 50, 100, and 150 for 8 h. **O** RT-qPCR was used to detect the expression of METTL3 and STAT5A in GC cells transfected with METTL3 siRNA prior to infecting with *H. pylori 26695* at a MOI of 100:1 for 8 h. **P** Western blot was used to detect the expression of STAT5A and METTL3 in the treated GC cells. The data are the means ± SD from three independent experiments. ns: no significance; *P < 0.05; **P < 0.01; ***P < 0.001; ****P < 0.0001.

To identify the downstream target genes of METTL3-mediated m^6^A modification, we performed MeRIP-Seq analysis to detect the differentially enriched genes upon METTL3 knockdown. Consistent with previous studies [17], the most prevalent m^6^A motif, GGACU, exhibited significant enrichment in both the negative control (NC) and METTL3 interference (si-METTL3) groups (Fig. S5A). Most of these m^6^A peaks were predominantly located within coding sequences (CDSs) and 3′ untranslated regions (UTRs), particularly near stop codon regions (Fig. S5B, C). KEGG pathway analysis of the differentially methylated genes revealed the enrichment of cancer pathway (Fig. S5 D), further confirming the correlation between METTL3-mediated m^6^A and the occurrence and progression of GC. To identify METTL3-mediated potential downstream target genes that promote GC progression, we conducted an intersection analysis between the genes with reduced m^6^A modifications upon METTL3 interference in the MeRIP-Seq assay and the differentially expressed genes identified in the GC datasets from TCGA and GSE54129 (Fig. 1A). Among all 44 MeRIP-seq peaks, five potential target genes (CDCP1, TBC1D7, LPIN2, VCAN, and STAT5A) exhibited significant differential expression in the TCGA and GEO datasets. We further used Kaplan‒Meier Plotter to analyze the correlation between the five potential target genes and patient prognosis. As shown in Fig. S6, patients with high expression of TBC1D7 and LPIN2 exhibited a favorable prognosis, while those with high expression of CDCP1, VCAN, and STAT5A exhibited a poor prognosis. It has been reported that METTL3 positively regulates CDCP1 and promotes the development of GC [31]. Therefore, we selected VCAN and STAT5A for further verification. Our RT-qPCR results showed that the average fold change of STAT5A was far greater than VCAN in response to METTL3 overexpression (Fig. 1B). Based on previous research reports, STAT5A, as a transcription factor, plays an important role in various aspects of cellular processes, such as proliferation, differentiation, survival and so on. It is also involved in a wide array of cellular signal transduction pathways and gene expression regulation. Moreover, correlation analysis of gastric tissues from the GEPIA database revealed that the expression of STAT5A was positively associated with the expression of METTL3 (Fig. 1C). Therefore, we focused on STAT5A for further investigation. We found that METTL3 knockdown resulted in a decrease in STAT5A mRNA in GC cell lines MKN-45 and SGC-7901 (Fig. 1D), while overexpression of WT-METTL3, but not the mutated METTL3, led to an increase in the mRNA level of STAT5A (Fig. 1E). Then, we detected whether the regulation of STAT5A mRNA by METTL3 occurs at the transcriptional level. We constructed a dual-luciferase reporter gene vector containing the STAT5A promoter fragment and transfected the plasmid together with the empty vector or METTL3 expression vector into MKN-45 cells. As shown in Fig. 1F, overexpression of WT-METTL3 or mutated METTL3 did not change the luciferase activity. Then, we further explored whether METTL3 affects the stability of STAT5A mRNA. The results showed that METTL3 overexpression significantly increased the stability of STAT5A mRNA and attenuated its degradation (Fig. 1G). Afterward, we performed western blot to investigate the regulatory effect of METTL3 on STAT5A at the protein level. We found that METTL3 knockdown significantly reduced the protein level of STAT5A (Fig. 1H), whereas the overexpression of WT-METTL3 significantly enhanced STAT5A protein expression. However, overexpression of the METTL3 mutant had no discernible effect on STAT5A expression (Fig. 1I). We further used STM2457, a small molecule inhibitor of METTL3, to treat GC cells and found that the expression of STAT5A was significantly inhibited by STM2457 (Fig. 1J, K). In addition, we found that there was a positive correlation between the expression of STAT5A and that of METTL3 in the GC tissue samples (Fig. 1L, M). To further explore the causal link between the *H.pylori* infection and METTL3 and STAT5A expression, we infected GC cells with *H. pylori* and found a dose-dependent increase in both METTL3 and STAT5A expression with escalating MOI of *H.pylori* (Fig. 1N). Additionally, we found that *H.pylori*-mediated up-regulation of STAT5A can be abolished by METTL3 knockdown, indicating that *H.pylori*-induced upregulation of STAT5A expression is mediated by METTL3 (Fig. 1O, P).

### METTL3 causes m^6^A modification within the CDS region of STAT5A

**Fig. 2:**
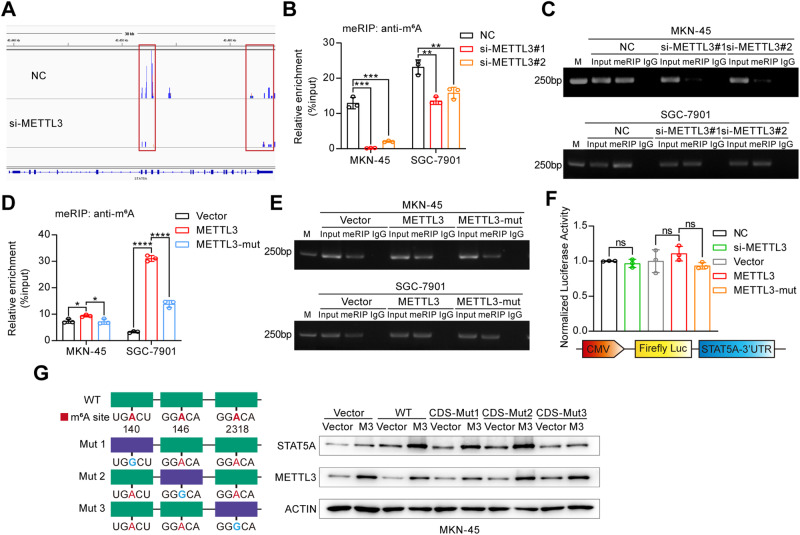
METTL3 causes the m^6^A modification of STAT5A at site A2318. **A** m^6^A modification peaks of STAT5A mRNA with significant differences between the si-METTL3 group and the NC group were visualized using Integrative Genomics Viewer (IGV) software. MeRIP-qPCR enrichment analysis of STAT5A using a m^6^A antibody in MKN-45 and SGC-7901 cells transfected with METTL3 siRNA (si-METTL3) (**B**) or METTL3 overexpression vector (**D**). The data are presented as the means ± SD from three independent experiments. Agarose gel electrophoresis analysis of qPCR products of STAT5A enriched through meRIP in MKN-45 and SGC-7901 cells with METTL3 knockdown (**C**) or overexpression (**E**). M: Molecular marker. **F** Dual-luciferase reporter gene assay was used to determine the effect of METTL3 knockdown or overexpression on the STAT5A 3′UTR. **G** Western blot analysis was performed to assess the expression of STAT5A in MKN-45 cells transfected with wild-type (WT) or the indicated m^6^A site-mutated STAT5A expression vector (CDS-Mut1, CDS-Mut2, or CDS-Mut3), together with an empty vector or a METTL3 expression vector. ns: no significance; *P < 0.05; **P < 0.01; ***P < 0.001; ****P < 0.0001.

Next, we explored whether METTL3 can cause m^6^A modification of STAT5A mRNA. We first analyzed the MeRIP-Seq data using Integrative Genomics Viewer (IGV) software and found that the m^6^A peak in the CDS and 3’ UTR of STAT5A mRNA decreased significantly following METTL3 knockdown (Fig. 2A). We further used MeRIP-qPCR to detect the effect of METTL3 on the m^6^A modification of STAT5A mRNA. The results showed that the knockdown of METTL3 resulted in a significant decrease in the m^6^A modification of STAT5A mRNA (Fig. 2B, C). In contrast, a significant increase in the m^6^A level of STAT5A was observed upon overexpression of WT-METTL3, but not the mutated METTL3 (Fig. 2D, E). To further identify the specific m^6^A site, we utilized the SRAMP (http://www.cuilab.cn/sramp) and RMBase 2.0 (https://rna.sysu.edu.cn/rmbase/index.php) platforms to predict potential m^6^A sites on STAT5A and identified five highly plausible sites (Fig. S7). Three of the predicted sites are within the CDS region (sites 140, 146, and 2318), while the remaining two are in the 3′UTR (sites 2959 and 3258). Accordingly, we performed a dual-luciferase reporter assay to assess whether the effect of METTL3 on the stability of STAT5A is achieved by causing m^6^A modification in the 3′UTR of STAT5A mRNA. Compared with that in the control group, the alteration in the expression level of METTL3 did not influence the luciferase reporter activity of STAT5A 3′UTR (Fig. 2F), indicating that METTL3 does not methylate the 3′UTR of STAT5A mRNA. To further evaluate whether METTL3 methylates STAT5A mRNA at the CDS region, we constructed different STAT5A expression vectors with predicted site mutations in the CDS region (Fig. 2G, left panel). GC cells were cotransfected with WT-STAT5A or different STAT5A mutants (CDS-Mut1-A140G, CDS-Mut2-A146G, and CDS-Mut3-A2318G), together with an empty vector or a METTL3 expression vector. Western blot results showed that METTL3 overexpression markedly increased the expression of STAT5A in MKN-45 cells transfected with the WT-STAT5A, CDS-Mut1, and CDS-Mut2 vectors, but had no significant effect on the expression of STAT5A in cells transfected with CDS-Mut3 (Fig. 2G, right panel), suggesting that the A2318 site in the STAT5A CDS region is a potential modification site.

### IGF2BP2 increases the stability of STAT5A mRNA in a m^6^A-dependent manner

**Fig. 3:**
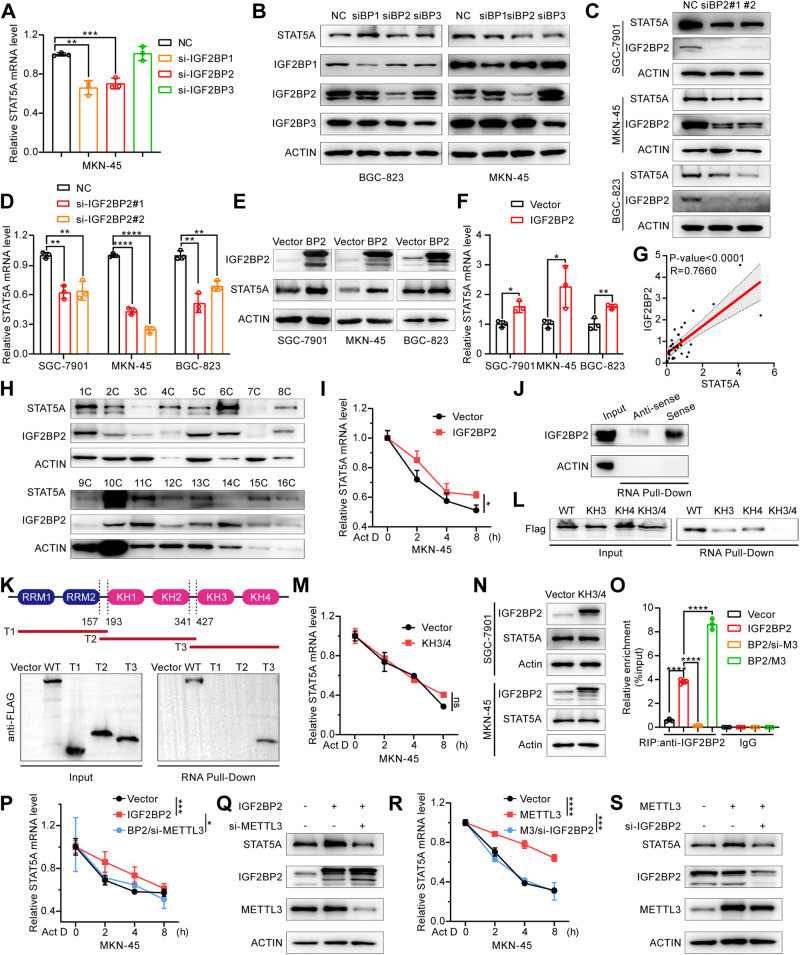
IGF2BP2 stabilizes STAT5A mRNA through its GXXG motif in a m^6^A-dependent manner. **A** The relative mRNA expression of STAT5A was assessed by qRT-PCR in MKN-45 cells with IGF2BP1/2/3 interference. **B** The expression levels of STAT5A were detected by Western blot in GC cells with IGF2BP1/2/3 knockdown. siBP1: IGF2BP1 siRNA; siBP2: IGF2BP2 siRNA; siBP3: IGF2BP3 siRNA. Western blot (**C**) and qRT-PCR (**D**) analyses of STAT5A expression in SGC-7901, MKN-45, and BGC-823 cells transfected with negative control (NC) or IGF2BP2 siRNAs (siBP2). Western blot (**E**) and qRT-PCR (**F**) analyses of STAT5A expression in SGC-7901, MKN-45, and BGC-823 cells transfected with an empty vector or an IGF2BP2 expression vector. BP2: IGF2BP2 overexpression. **G** Correlation analysis of IGF2BP2 and STAT5A protein expression in GC tissues from 35 patients. The data comes from the ratio of the western blot band density of IGF2BP2 and STAT5A to the corresponding β-actin. **H** Representative Western blot results of STAT5A and IGF2BP2 expression in GC tissues. **I** RT-qPCR analysis of STAT5A mRNA level in MKN-45 cells transfected with empty vector or the IGF2BP2 expression vector prior to treating with Act D for the indicated time points. **J** IGF2BP2 was identified by immunoblotting analysis in MKN-45 cells subjected to an RNA pull-down assay using sense or antisense STAT5A probes. **K** RNA pull-down assay was used to detect the interaction between STAT5A mRNA and truncated IGF2BP2 by adding the STAT5A sense probe to GC cells transfected with a Flag-tagged wild-type (WT) or truncated IGF2BP2 expression vector (T1, T2, T3). The pulled-down protein was subjected to Western blot analysis. **L** RNA pull-down assay was performed to detect the direct interaction between STAT5A mRNA and the GXXG motif of IGF2BP2 in MKN-45 cells transfected with KH3 or KH4 domain mutants or a double mutant (KH3/4) of IGF2BP2 in which the GXXG motif in KH3 or KH4 or both was mutated. **M** The decay rate of STAT5A mRNA was determined by RT-qPCR in MKN-45 cells transfected with the IGF2BP2 KH3/4 mutant or empty vector prior to treatment with Act D for the indicated times. KH3/4: IGF2BP2 expression vector with GXXG motif mutations in both the KH3 and KH4 domains. **N** Western blot analysis of the expression of STAT5A in SGC-7901 and MKN-45 cells transfected with the IGF2BP2 KH3/4 mutant or empty vector. **O** The effect of METTL3 on the direct interaction between the IGF2BP2 protein and STAT5A mRNA was determined with a RIP assay by adding an anti-IGF2BP2 antibody to MKN-45 cells cotransfected with the IGF2BP2 expression vector together with METTL3 siRNA or METTL3 expression vector. BP2/si-M3: IGF2BP2 overexpression & METTL3 siRNA; BP2/M3: IGF2BP2 overexpression & METTL3 overexpression. **P**, **R** The relative STAT5A mRNA expression was analyzed using qRT-PCR in MKN-45 cells transfected with the indicated vector or siRNA prior to adding Act D for 0, 2, 4, or 8 h to detect the stability of STAT5A mRNA. BP2: IGF2BP2 expression vector; M3: METTL3 expression vector. **Q**, **S** Western blot was used to analyze the protein expression levels of STAT5A in different transfected GC cell lines. The data are presented as the means ± SD from three independent experiments. ns: no significance; *P < 0.05; **P < 0.01; ***P < 0.001; ****P < 0.0001.

IGF2BPs (IGF2BP1/2/3) constitute a distinct group of m^6^A readers that specifically recognize the GG(m^6^A)C motif within mRNA transcripts [32] and play a pivotal role in augmenting the stability of targeted mRNAs through a m^6^A-dependent mechanism [24]. Considering that METTL3-mediated m^6^A modification enhances STAT5A mRNA stability, we speculated on the potential involvement of IGF2BPs as readers of m^6^A-modified STAT5A. We knocked down IGF2BP1/2/3 to evaluate the impact on STAT5A expression using RT-qPCR and Western blot analysis. Our results showed that compared with that of IGF2BP1 and IGF2BP3, targeted knockdown of IGF2BP2 resulted in a more significant reduction in STAT5A expression at both the mRNA and protein levels (Fig. 3A, B). Then, we synthesized another IGF2BP2 siRNA and confirmed that IGF2BP2 siRNAs significantly reduced STAT5A mRNA and protein levels in different GC cell lines (Fig. 3C, D, and Fig. S8A). In contrast, the overexpression of IGF2BP2 significantly increased STAT5A mRNA and protein levels in the GC cell lines (Fig. 3E, F, and Fig. S8B). In the tissue samples of GC patients, the expression of STAT5A was significantly positively correlated with the expression of IGF2BP2 (Fig. 3G. H). Subsequently, we detected whether IGF2BP2 affects STAT5A mRNA stability. Our results demonstrated that IGF2BP2 overexpression significantly increased the half-life of STAT5A mRNA (Fig. 3I). A previous study showed that YTHDF2 can recognize STAT5A and mediate its degradation in multiple myeloma [33]. Therefore, we tried to investigate whether YTHDF2 has a regulatory effect on the expression of STAT5A in GC cells. Our results showed that overexpression or knockdown of YTHDF2 did not significantly affect the expression of STAT5A in GC cells (Fig. S9A–D), suggesting that the regulation of STAT5A expression may be influenced by different readers in different cell lines. To further investigate the interaction between STAT5A mRNA and IGF2BP2, we performed an RNA pull-down assay using a STAT5A probe. As shown in Fig. 3J, IGF2BP2 was pulled down by the STAT5A sense probe, but not the antisense probe, indicating the specific binding of STAT5A mRNA to IGF2BP2. To further investigate the specific IGF2BP2 domains that bind to STAT5A mRNA, we constructed several Flag-tagged WT and truncated IGF2BP2 expression vectors (Fig. 3K), which were subsequently transfected into MKN-45 cells for RNA pull-down assays. The results showed that Flag-tagged WT-IGF2BP2 and the truncated IGF2BP2 mutant containing the KH3 and KH4 domains (T3 mutant) could be pulled down by the STAT5A probe, whereas the truncated mutant of IGF2BP2 containing either the RRM1-2 domain (T1 mutant) or KH1-2 domain (T2 mutant) could not be pulled down (Fig. 3K), indicating that the KH3 and KH4 domains of IGF2BP2 can selectively bind to STAT5A mRNA. Furthermore, the GXXG motif in the KH3 and KH4 domains of IGF2BP2 has been reported to play a key role in recognition and interaction with target mRNAs [34]. Therefore, to determine whether the GXXG motifs in IGF2BP2 participate in mediating interactions with STAT5A, we introduced a GDDG mutation into the GXXG motif of either the KH3 or KH4 domain of IGF2BP2, as well as both the KH3 and KH4 domains, followed by an RNA pull-down assay. As shown in Fig. 3L, a single mutation in the KH3 or KH4 domain partially abolished the interaction between STAT5A mRNA and IGF2BP2, and double mutations in the GXXG motif within the KH3 and KH4 domains of IGF2BP2 completely eliminated this interaction, indicating that the GXXG motifs in both the KH3 and KH4 domains mediated the interaction between STAT5A mRNA and IGF2BP2. We further transfected the IGF2BP2 KH3 and KH4 mutant into GC cells to determine whether the IGF2BP2 KH3 and KH4 mutant can affect the mRNA stability and protein level of STAT5A. As shown in Fig. 3M, N, the overexpression of the IGF2BP2 KH3 and KH4 mutant no longer affected the mRNA stability and protein expression of STAT5A.

Subsequently, to further investigate whether the m^6^A modification of STAT5A mRNA is required for the interaction between IGF2BP2 and STAT5A mRNA, we performed RIP-qPCR experiments using an IGF2BP2 antibody in MKN-45 cells overexpressing IGF2BP2, followed by either METTL3 knockdown or overexpression. We observed that IGF2BP2 overexpression enhanced the interaction between IGF2BP2 and STAT5A mRNA. However, METTL3 knockdown significantly attenuated this interaction, and METTL3 overexpression substantially augmented this interaction (Fig. 3O), indicating that METTL3-mediated m^6^A methylation is crucial for the interaction between IGF2BP2 and STAT5A mRNA. Then, we knocked down METTL3 in GC cells overexpressing IGF2BP2 and detected STAT5A mRNA stability and protein expression. As shown in Fig. 3P, Q, METTL3 knockdown significantly abolished the IGF2BP2-mediated increase in STAT5A mRNA stability and protein expression. We further knocked down IGF2BP2 in GC cells overexpressing METTL3 and detected STAT5A mRNA stability and protein expression. As expected, the knockdown of IGF2BP2 also abrogated the METTL3-mediated increase in STAT5A mRNA stability and protein expression (Fig. 3R, S). These results suggest that both METTL3-mediated m^6^A modification and the recognition and binding of IGF2BP2 to the STAT5A m^6^A site are crucial for the mRNA stability and protein expression of STAT5A.

### STAT5A functions as an oncogene in gastric carcinoma

**Fig. 4:**
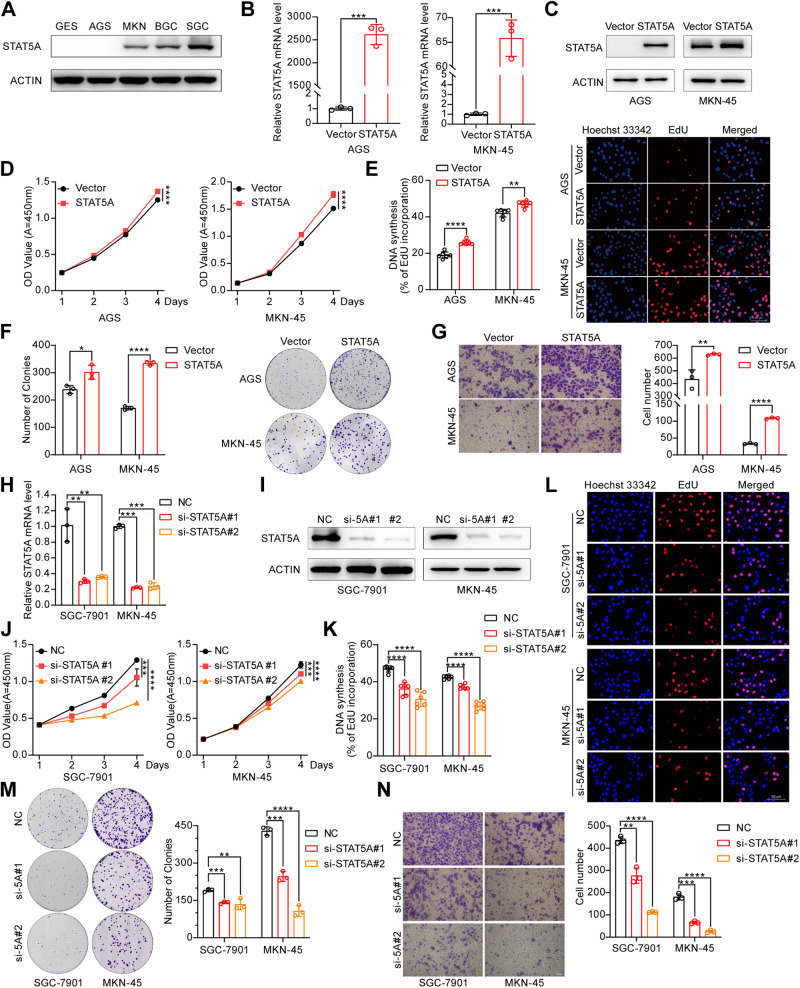
STAT5A increases GC cell proliferation and migration in vitro. **A** Western blot analysis of STAT5A expression in immortalized GES-1 cells and different GC cell lines. Overexpression of STAT5A mRNA and protein was verified by RT-qPCR (**B**) and Western blot (**C**) in AGS and MKN-45 cells transfected with a STAT5A expression vector (STAT5A). The effect of STAT5A overexpression on GC cell proliferation was assessed by the CCK8 assay (**D**), EdU assay (**E**), and colony formation assay (**F**). The left panels of **E** and **F** show the statistical analysis results. The right panels show representative images. **G** Effect of STAT5A overexpression on GC cell migration. The left panel shows representative images. The right panel shows the statistical analysis results. The efficiency of STAT5A knockdown was verified by RT-qPCR (**H**) and Western blot (**I**) in GC cells transfected with STAT5A siRNA. si-5A: STAT5A siRNA. The effect of STAT5A knockdown on GC cell proliferation was assessed by a CCK-8 assay (**J**), an EdU assay (**K**, **L**), and a colony formation assay (**M**). **N** The Transwell assay in GC cells transfected with NC or STAT5A siRNA. The left panel shows representative images. The right panel shows the statistical analysis results. si-5A: STAT5A siRNA. The data are presented as the means ± SD from three independent experiments. *P < 0.05; **P < 0.01; ***P < 0.001; ****P < 0.0001.

To further investigate the function of STAT5A in GC, we first used Western blot to determine STAT5A expression in the gastric immortalized epithelial cell line GES-1 and four GC cell lines, AGS, BGC-823, MKN-45, and SGC-7901. The results showed that the protein expression of STAT5A in immortalized GES-1 cells and AGS cells was not detected by Western blot, while it was relatively high in SGC-7901 cells and showed a moderate expression level in MKN-45 cells (Fig. 4A). Therefore, STAT5A was overexpressed in AGS and MKN-45 cells, while STAT5A was knocked down in MKN-45 and SGC-7901 cells. RT-qPCR and Western blot verified the overexpression efficiency (Fig. 4B, C). The results of cell proliferation experiments, including CCK-8, EdU, and colony formation assays, as well as Transwell cell migration experiment indicated that the overexpression of STAT5A in AGS and MKN-45 cells significantly promoted cell proliferation (Fig. 4D–F) and cell migration (Fig. 4 G). In contrast, STAT5A knockdown with two distinct siRNAs inhibited cell proliferation and migration in the SGC-7901 and MKN-45 cell lines (Fig. 4H–N). These findings provide evidence for the oncogenic role of STAT5A in GC progression.

### STAT5A knockdown inhibits tumor growth in vivo

**Fig. 5:**
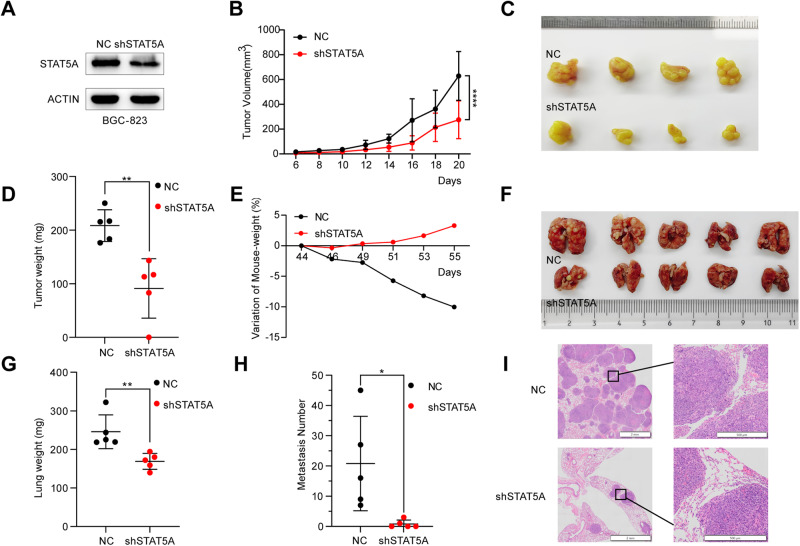
Knockdown of STAT5A inhibits the growth and metastasis of GC cells in vivo. **A** Stable knockdown of STAT5A in BGC-823 cells was verified by western blot. **B** The tumors volume in the xenograft mice was monitored every 2 days from day 6 to day 20 after subcutaneous injection of BGC-823 cells containing sh-NC or sh-STAT5A. **C** The subcutaneous tumor in nude mice injected with BGC-823 cells containing sh-NC or sh-STAT5A. **D** Statistical analysis was performed on the subcutaneous tumor mass, which is presented as the means ± SD from the samples of 5 mice. **E** The body weights of the nude mice were monitored every 2 days from day 44 to day 55 after injecting BGC-823 cells harboring negative control or sh-STAT5A from the tail vein. **F** The mice were euthanized on the 55^th^ day after tail vein injection. The lung tissues of the mice are shown. **G** Statistical analysis of the lung weight in (**F**). **H** Statistical analysis of the number of lung metastatic nodules in (**F**). **I** Lung tissues were subjected to H&E staining for the detection of metastatic nodules. *P < 0.05; **P < 0.01; ****P < 0.0001.

To further characterize the oncogenic role of STAT5A in GC, we conducted a subcutaneous tumor formation experiment in nude mice to assess the impact of STAT5A knockdown on the tumorigenesis ability of GC cells in vivo. BGC-823 cells were infected with lentivirus harboring shRNA targeting STAT5A or its corresponding negative control, and stable knockdown cells were screened and identified (Fig. 5A). The xenografts in the NC group exhibited significantly greater volume and weight than those in the STAT5A knockdown group (Fig. 5B-D). Subsequently, to evaluate the impact of STAT5A on the metastatic potential of GC cells, we conducted a tail vein injection experiment by separately injecting cells of the control group and the shSTAT5A group into the tail vein of nude mice. The mice in the shSTAT5A group exhibited a consistent upward trend in weight by the 55th day, whereas those in the control group displayed a significant decrease starting from the 44th day in weight (Fig. 5E). In addition, both the size and weight of the lungs of the mice injected with shSTAT5A cells were significantly lower than those injected with control cells (Fig. 5F, G). Furthermore, compared with the control group, the STAT5A knockdown group exhibited significantly fewer lung metastasis nodules (Fig. 5H). HE staining assay confirmed the presence of solid tumors in the lung of the mice (Fig. 5I). In conclusion, the growth of GC cells was significantly inhibited by stable knockdown of STAT5A, and the incidence of lung metastasis was significantly decreased in the shSTAT5A group. Thus, these findings provide evidence for the oncogenic role of STAT5A in GC tumorigenesis and metastasis.

### STAT5A is involved in METTL3-mediated biological functions

**Fig. 6:**
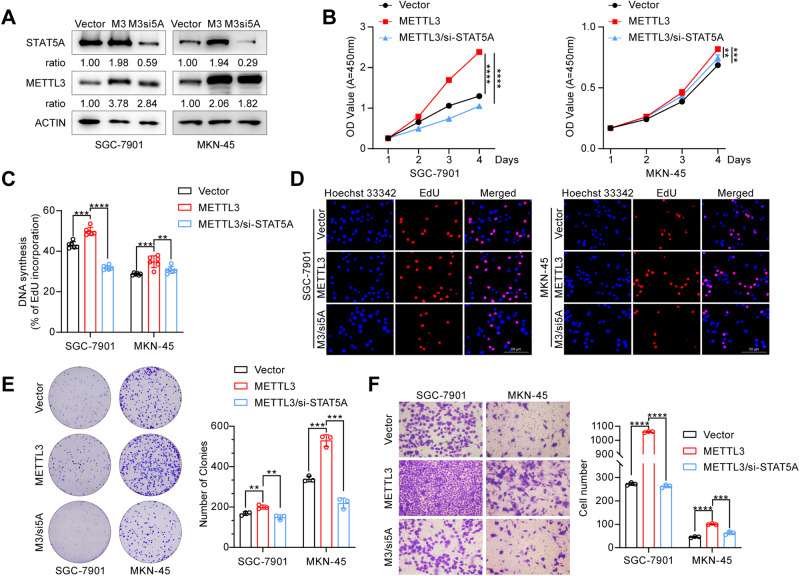
STAT5A knockdown abrogates METTL3-mediated biological function in GC cells. **A** Western blot was used to analyze the expression of METTL3 and STAT5A in different transfected GC cell lines. M3: METTL3 overexpression; M3si5A: METTL3 overexpression & STAT5A siRNA. **B** CCK-8 assay was used to determine the proliferation of SGC-7901 and MKN-45 cells with different transfections. METTL3: METTL3 overexpression; si-STAT5A: STAT5A siRNA. Statistical analysis (**C**) and representative images (**D**) of EdU assay in different transfected GC cells. M3/si5A: METTL3 overexpression & STAT5A siRNA. **E** Assessment of colony formation ability in treated GC cells. Left: representative images; Right: Statistical analysis results. **F** Transwell migration analysis was performed in SGC-7901 and MKN-45 cells with different transfections. Representative images of migrated cells are shown (left). The number of migrated cells was quantified (right). The data are presented as the means ± SD from three independent experiments. **P < 0.01; ***P < 0.001; ****P < 0.0001.

We then asked whether STAT5A is required for METTL3-mediated biological functions. We cotransfected the METTL3 expression vector, together with control siRNA or STAT5A siRNA, into the SGC-7901 and MKN-45 cell lines (Fig. 6A) and then performed functional restoration assays. The results showed that the overexpression of METTL3 led to a significant increase in the proliferation and migration ability of SGC-7901 and MKN-45 cells, which was subsequently abolished upon the depletion of STAT5A (Fig. 6B–F).

### STAT5A binds to the promoter of KLF4 and regulates KLF4 expression

**Fig. 7:**
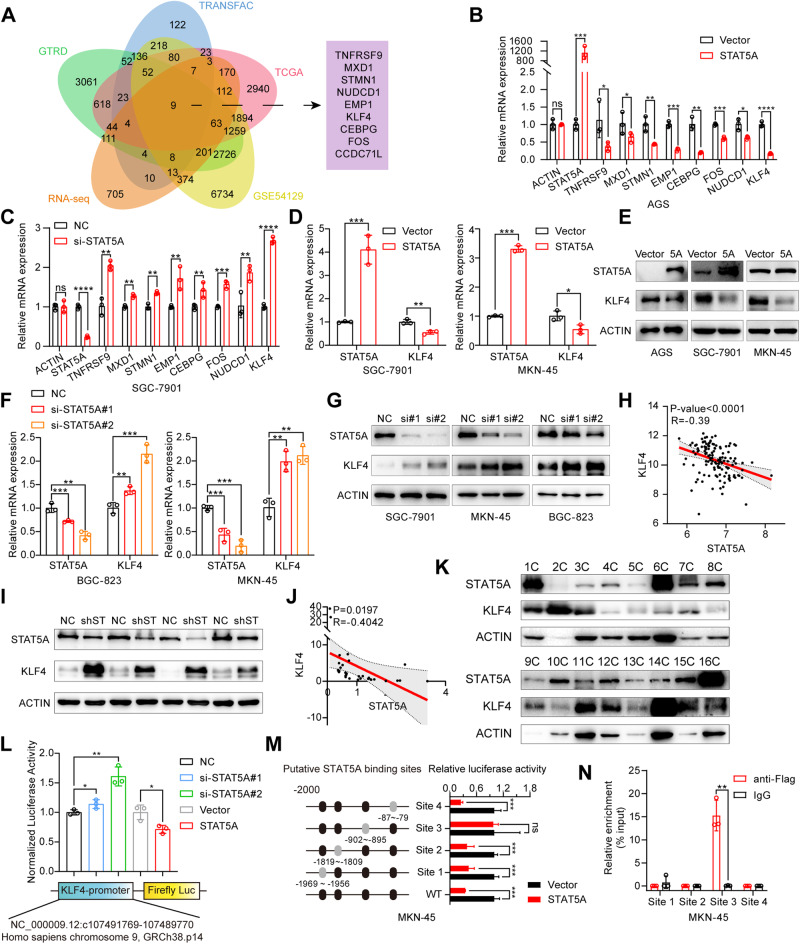
KLF4 is a crucial downstream target of STAT5A. **A** Venn diagram to show 9 potential downstream target genes that may be regulated by STAT5A by intersecting 5 high-throughput analyses. RNA-seq: differentially expressed genes detected by RNA-seq in AGS cells transfected with STAT5A expression vector. TRANSFAC and GTRD: prediction of the downstream target genes that may be regulated by STAT5A. TCGA and GSE54129: differentially expressed genes in GC tissues according to the data from TCGA and GEO databases. **B** The relative mRNA expression levels of candidate STAT5A target genes were assessed by RT-qPCR in AGS cells transfected with either the empty vector or STAT5A expression vector. **C** The relative mRNA expression levels of candidate STAT5A target genes were measured by RT-qPCR in SGC-7901 cells transfected with NC or STAT5A siRNA. **D** RT-qPCR was used to detect the expression of KLF4 in BGC-823 and MKN-45 cells transfected with either the empty vector or STAT5A expression vector. **E** The expression of KLF4 in GC cells transfected with either the empty vector or STAT5A expression vector was detected by Western blot. 5A: STAT5A overexpression. The expression of KLF4 was assessed using RT-qPCR (**F**) and Western blot (**G**) assays in GC cells with STAT5A knockdown. si:STAT5A siRNA. **H** Correlation between KLF4 and STAT5A mRNA in GC patients from GEO (GSE54129) database. **I** Western blot analysis of the expression of STAT5A and KLF4 in the tumor tissues of Fig. 5C. shST: Sh-STAT5A. **J** Correlation analysis of KLF4 and STAT5A protein in GC tissues from 35 patients. The data comes from the ratio of the western blot band density of KLF4 and STAT5A to the corresponding β-actin. **K** Representative western blot results of STAT5A and KLF4 expression in GC tissues. **L** Dual-luciferase reporter gene assay detection in MKN-45 cells transfected with the pGL3-KLF4 promoter vector and pRL-TK vector, together with STAT5A siRNA or the STAT5A overexpression vector. **M** The schematic diagram on the left illustrates the mutations of four putative STAT5A binding regions in the KLF4 promoter predicted by the contraV3 platform (bioit2.irc.ugent.be/contra/v3/). The right image shows the dual-luciferase reporter gene activity in GC cells transfected with the KLF4 wild-type or mutant promoter luciferase vector and pRL-TK, together with the empty vector or the STAT5A expression vector. **N** The binding of STAT5A to site 3 (−902 bp~−895 bp) of the KLF4 promoter was validated through ChIP-qPCR analysis. The data are presented as the means ± SD from three independent experiments. ns: no significance; *P < 0.05; **P < 0.01; *** *P* < 0.001; **** *P* < 0.0001.

To further explore the downstream targets of STAT5A in GC, we performed RNA-seq in AGS cells transfected with the STAT5A expression vector or the empty vector. The results revealed that a total of 1838 genes exhibited significant differential expression following STAT5A overexpression in AGS cells (Fig. S10A). Subsequent enrichment analysis using GO, KEGG, and Reactome pathway gene sets demonstrated that these genes were closely associated with cell division and the cell cycle (Fig. S10B–D). Given that STAT5A acts as a transcription factor [35], we utilized the TRANSFAC and GTRD platforms to predict potential downstream genes regulated by STAT5A. At the same time, we screened the differentially expressed genes in GC tissues from the TCGA and GEO (GSE54129) databases. We intersected the RNA-seq data and the data from TRANSFAC, GTRD, TCGA, and GEO databases and identified 9 candidate genes (Fig. 7 A). Since there are almost no reports on the correlation between the CCDC71L gene and cancer in the published literature, we conducted RT-qPCR to detect the expression of 8 other potential downstream genes following STAT5A overexpression in AGS cells or STAT5A interference in SGC-7901 cells. Among the candidate genes, KLF4 showed the most significant change upon STAT5A overexpression or knockdown (Fig. 7B, C). We further validated the regulatory effect of STAT5A on KLF4 at both the mRNA and protein levels in different GC cell lines. Our results showed that overexpression of STAT5A resulted in a significant decrease in the expression of KLF4 (Fig. 7D, E), while knockdown of STAT5A led to increased expression of KLF4 in GC cells (Fig. 7F, G). Additionally, in the GEO dataset (GSE54129), a significant inverse correlation was observed between the expression levels of KLF4 and STAT5A (Fig. 7H). We further detected the expression level of STAT5A and KLF4 in the subcutaneous tumor tissue of nude mice in Fig. 5C. We found a significant decrease in the protein expression of STAT5A and a significant increase in the protein expression of KLF4 in the tumors of the shSTAT5A group compared to those of the control group, confirming the negative regulatory effect of STAT5A on KLF4 in these tissues (Fig. 7I). Furthermore, in the tissue samples of patients with GC, we also confirmed that the protein level of STAT5A was negatively correlated with that of KLF4 (Fig. 7J, K). To further investigate whether the regulation of KLF4 by STAT5A occurs at the transcriptional level, we constructed a KLF4 promoter luciferase reporter vector and performed a dual-luciferase reporter assay. As shown in Fig. 7L, STAT5A knockdown increased luciferase activity of KLF4 promoter, whereas STAT5A overexpression decreased luciferase activity. According to the ConTraV3 website prediction, we introduced mutations within the predicted high-confidence STAT5A binding sites of the KLF4 promoter. We cotransfected these mutant vectors with an empty vector or a STAT5A expression vector into GC cells and performed a dual-luciferase reporter assay. We observed that overexpression of STAT5A did not significantly alter the dual luciferase activity of mutant site 3 (−902 to −895) (Fig. 7M). To further confirm the luciferase activity results, we performed a ChIP assay. Consistent with the results of the luciferase activity assay, ChIP-qPCR revealed greater enrichment of DNA fragments at site 3 in MKN-45 cells overexpressing Flag-STAT5A (Fig. 7N). These findings suggest that site 3 may function as the putative binding region for STAT5A on the KLF4 promoter. The Tyr-694 site of STAT5A is the key site for its phosphorylation and dimerization [36]. Therefore, we constructed a non-phosphorylatable mutation of STAT5A (Y694A) [36], which fails to form an activated dimer, and detected the regulatory effect of the mutated STAT5A (Y694A) on the expression of KLF4. Our results showed that STAT5A (Y694A) can not abrogate the inhibitory effect on KLF4 (Fig. S11), indicating that the phosphorylation or dimerization of STAT5A is not necessary for regulating KLF4 expression.

### STAT5A exerts its biological functions partially through KLF4

**Fig. 8:**
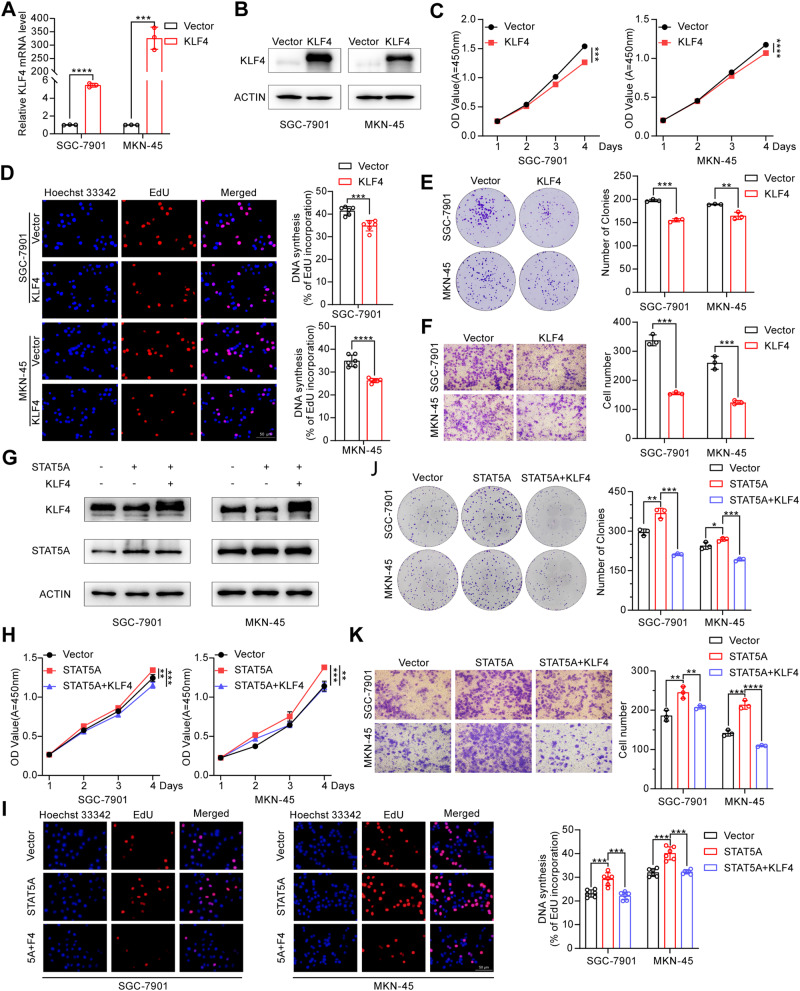
KLF4 overexpression partially abolishes STAT5A-mediated biological effects in vitro. RT-qPCR (**A**) and Western blot (**B**) analyses of relative KLF4 expression in SGC-7901 and MKN-45 cells transfected with KLF4. CCK-8 (**C**), EdU (**D**), and colony formation (**E**) assays were performed to evaluate the proliferation capacity of GC cells transfected with the KLF4 expression vector. Left panels of (**D**) and (**E**): representative images. Right panel: Statistical analysis results. **F** The migration ability of GC cells transfected with KLF4 or empty vector was assessed using a Transwell assay. Left panel: representative images. Right panel: Statistical analysis. **G** Western blot analysis of the expression of KLF4 and STAT5A in different transfected SGC-7901 and MKN-45 cells. The biological effect of STAT5A on cell proliferation and cell migration was effectively reversed by cotransfection of KLF4 and STAT5A expression vectors into GC cells, as determined by CCK-8 (**H**), EdU (**I**), colony formation (**J**), and Transwell (**K**) experiments. The left panels of (**I**), (**J**), and (**K**) are representative images. Right panel: Statistical analysis. 5A + F4 in (**I**): STAT5A and KLF4 overexpression. The data are presented as the means ± SD from three independent experiments. *P < 0.05; **P < 0.01; ***P < 0.001; ****P < 0.0001.

Having revealed the regulatory effect of STAT5A on KLF4, we next aimed to investigate whether STAT5A exerts its biological effect through KLF4. We first investigated whether KLF4 regulates cell proliferation and migration in cancer cells. As shown in Fig. 8A–F, overexpression of KLF4 inhibited GC cell proliferation and migration in vitro. Subsequently, we conducted functional recovery experiments by overexpressing STAT5A and KLF4 in SGC-7901 and MKN-45 cells (Fig. 8G). Our study demonstrated that the enforced expression of KLF4 can counterbalance the oncogenic impact of STAT5A (Fig.8 H–K).

## Discussion

GC was one of the most prevalent malignancies globally. *H. pylori* infection is one of the most important environmental factors contributing to the malignant transformation of gastric mucosal epithelial cells [37]. In this study, we found that infection with *H. pylori* increases the expression of METTL3 in GC cells, which is consistent with a recent report that infection with *H. pylori* can result in the upregulation of METTL3 in mouse models [38]. Accumulating evidence in recent years has demonstrated that METTL3 functions as an oncogene and plays an essential role in tumorigenesis and progression [39, 40]. It has been reported that METTL3 promotes GC progression by binding to PABPC1 and stabilizing its target mRNA [41] or mediating the m^6^A modification of several genes, such as HDGF [29], ZMYM1 [30], and NDUFA4 [42]. However, the m^6^A modification of other important downstream target molecules regulated by METTL3 in GC is still not fully understood. In this study, we performed MeRIP-Seq in GC cells with METTL3 knockdown and screened 44 differential enriched genes. We intersected the MeRIP-Seq results with the differentially expressed genes predicted by the TCGA and GEO databases in GC and identified 5 candidate genes (CDCP1, TBC1D7, LPIN2, VCAN, and STAT5A). Given that TBC1D7 and LPIN2 were associated with favorable prognosis which is divergent with their upregulated expression in GC tissues and CDCP1 had been reported to be regulated by METTL3, we selected VCAN and STAT5A for further verification. Our results indicated that the average fold change of STAT5A was far greater than VCAN in response to METTL3 overexpression. In addition, we found that STAT5A plays a more pivotal role in cancer progression and patient prognosis based on previous studies. Therefore, we focused on STAT5A for further investigation. We further verified that STAT5A is a bona fide downstream molecule modified by METTL3 in GC and that A2318 on STAT5A mRNA is the m^6^A site. To the best of our knowledge, this is the first report on the m^6^A modification of STAT5A by METTL3 in GC.

STAT5A is a member of the signal transducers and activators of the transcription (STAT) protein family and participates in the JAK/STAT signaling pathway [43]. The JAK/STAT signaling pathway regulates multiple crucial cellular processes, including cell proliferation, stemness self-renewal, and the immune response [44, 45]. Several studies have indicated crosstalk between m^6^A and the JAK-STAT pathway. METTL3 enhances ribosomal translation efficiency through the METTL3/m^6^A/JAK1/STAT3 axis and facilitates tumor immune evasion [46]. In addition, it has been suggested that decreased METTL3 expression increases SOCS stability, thereby inhibiting IL7-STAT5 signaling pathway activation, which further leads to abnormalities in T-cell proliferation and differentiation [47]. However, few studies have explored the role and mechanism of the m^6^A modification of JAK-STAT pathway members on the occurrence and development of GC [48]. Our research provides evidence that METTL3-mediated m^6^A modification of STAT5A increases the stability and expression of STAT5A. Moreover, STAT5A plays an oncogenic role in GC by promoting cell proliferation and metastasis.

m^6^A-modified mRNAs can be recognized and bound by different m^6^A readers. m^6^A readers can be divided into three major classes: IGF2BPs, YT521-B homology (YTH) domain family proteins, and a set of heterogeneous nuclear ribonucleoproteins (hnRNPs) [15, 49, 50]. IGF2BPs can promote the stability and translation of m^6^A-modified RNAs; YTH domain family proteins mainly regulate target mRNA splicing, nuclear export, and translation, while hnRNPs can remodel local RNA structures and regulate alternative splicing or the processing of target transcripts [13, 15, 25]. In this study, our RNA pull-down and RIP assays results verified that STAT5A mRNA can be recognized and bound by IGF2BP2. The GXXG motif in the KH3 and KH4 domains of IGF2BP2 is essential for its recognition and interaction with STAT5A. Furthermore, METTL3 and IGF2BP2 are interdependent and cooperate to regulate STAT5A expression. It has been reported that YTHDF2 recognizes m^6^A-modified STAT5A and promotes its mRNA degradation in multiple myeloma [33]. However, we didn’t find any regulatory effect of YTHDF2 on STAT5A expression in the GC cells. These distinct findings suggest that STAT5A exhibits heterogeneity across different types of cancers, suggesting that m^6^A-modified STAT5A may interact with distinct reader proteins, thereby leading to diverse outcomes. It has been reported that other genes may also be recognized by different readers in different types of tumors. For example, in glioblastoma stem cells, m^6^A-modified MYC is recognized by YTHDF2 [51], while in acute myeloid leukemia, it is recognized by IGF2BP2 [52]. This discrepancy suggests a complicated regulatory network underlying the role of m^6^A modification in regulating tumor progression, which needs to be further clarified. Further investigation is also required to elucidate the underlying mechanisms governing the preferential recruitment of distinct readers to the same mRNA in diverse cancer types or tissues.

STAT5A affects cancer progression through multiple mechanisms, such as cell proliferation, differentiation, apoptosis, hematopoiesis, and immunity [45, 53–55]. It has been reported that STAT5A binds to the CD44 promoter to promote its transcription [56] or hinders DNA damage repair by inhibiting RAD51 [57], thereby promoting the occurrence and development of GC. In this study, we confirmed that KLF4 was a bona fide downstream molecule regulated by STAT5A in GC cells. STAT5A can directly bind to the −902 to −895 region of the KLF4 promoter, thereby inhibiting KLF4 expression. Furthermore, STAT5A-mediated biological functions can be partially abrogated by KLF4 overexpression. As a transcription factor, STAT5A may regulate many other target genes. We noticed that two well-known tumor suppressor genes, TNFRSF9 (pivotal immune checkpoint molecules) [55] and MXD1 (a component of a transcriptional repressor complex) [6, 56] also exhibited notable decreases in expression following STAT5A overexpression, suggesting that STAT5A may exert its oncogenic function partially through these molecules, which need to be further investigated.

As a zinc finger transcription factor, KLF4 was initially identified in the differentiated epithelium of the colon and small intestine of neonatal mice in 1996. Substantial evidence has shown that KLF4 plays an important regulatory role in cell proliferation and differentiation [58]. The dysregulation of KLF4 is associated with the occurrence and development of tumors. However, The mechanism of KLF4 dysregulation in tumors is not fully understood. It has been reported that METTL3 can cause the m^6^A modification of KLF4 mRNAs in bladder cancer [58]. To determine whether KLF4 mRNA was directly regulated by MTTL3-mediated m^6^A methylation in GC, we performed MeRIP-qPCR to detect the effect of METTL3 overexpression on the m^6^A modification of KLF4 mRNA. The results showed that METTL3 overexpression has no significant effect on the m^6^A modification of KLF4 mRNA, which excludes the possibility that METTL3 directly regulates KLF4 mRNA in GC (Fig. S12A, B). Therefore, our findings that KLF4 can be directly inhibited by STAT5A at the transcriptional level revealed a novel mechanism to explain the dysregulation of KLF4 in GC.

**Fig. 9:**
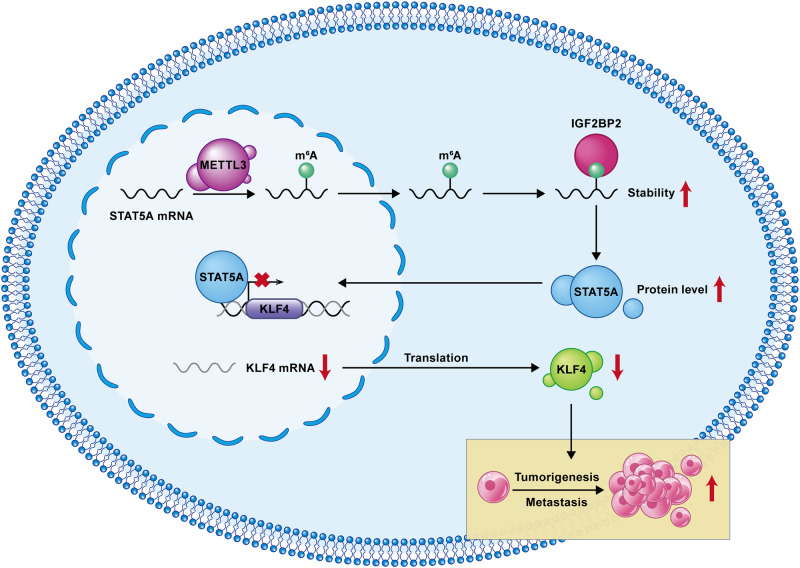
The working model in this study. METTL3 is responsible for m^6^A modification of STAT5A mRNA. m^6^A on STAT5A can be recognized and bound by IGF2BP2, thereby increasing the stability and expression of STAT5A. STAT5A promotes GC proliferation and metastasis by binding to the KLF4 promoter and inhibiting its expression.

In summary, we confirmed that METTL3 mediated STAT5A mRNA m^6^A modification at the A2318 site. The m^6^A modification of STAT5A can be recognized and bound by the GXXG motif in the KH3/4 domains of IGF2BP2. The combination of m^6^A modification and the binding of IGF2BP2 to the m^6^A site contributes to the increased stability of STAT5A mRNA and protein expression in GC. STAT5A exerts its oncogenic function by suppressing the transcription of KLF4. Moreover, increasing the expression of KLF4 can efficiently antagonize the biological function of STAT5A in GC (Fig. 9). Therefore, our findings provide a strategic framework for integrating clinical, molecular, and genomic profiles, facilitating the optimization of diagnostic accuracy and therapeutic strategies for GC.

## Materials and methods

### Cell culture and inhibitor treatment

The gastric immortalized epithelial cell line GES-1 and the GC cell lines AGS, BGC-823, MKN-45, and SGC-7901 were stored in our laboratory. GES-1, MKN-45, BGC-823, and SGC-7901 cells were conventionally maintained in RPIM-1640 medium (Biological Industries, Cat# 01-100-1A) supplemented with 10% fetal bovine serum (Pricella, Cat# 164210-50) and 1% penicillin–streptomycin (Beyotime, Cat# C0222). AGS cells were cultured in F-12 medium (Macgene, Cat# CM10080) supplemented with 10% fetal bovine serum and 1% penicillin–streptomycin. All the cells were cultured at 37 °C in a humidified incubator with 5% CO_2_.

The GC cells were treated with either DMSO or 10 μM STM2457 (MCE, Cat# HY-134836), a specific inhibitor of METTL3 enzyme activity, for 72 h. Then, the treated cells were harvested for subsequent analysis.

### siRNAs and plasmids

The small interfering RNAs (siRNAs) were synthesized from Genepharma (Shanghai, China). The sequences of the siRNAs used are listed in Table S1. The siRNAs were transfected into the GC cells using Lipofectamine 2000 (Invitrogen, Cat# 11668019) according to the manufacturer’s instructions.

The pcDNA3.1-METTL3 plasmid was purchased from Addgene (Cat# 53739). pEnCMV-IGF2BP2-3×FLAG (Cat# P51857), pCMV-KLF4-3×FLAG (Cat# P46930) and pCMV-YTHDF2-3×Myc-SV40-Neo (Cat# P35696) were purchased from MIAOLING BIOLOGY. The pENTER-STAT5A-Flag&6×His plasmid was purchased from WZ Biosciences, Inc. (Cat# CH854425). The mutant plasmids were generated using PCR-based methods with designed primers (Table S2) and cloned into the vector with a ClonExpress Ultra One Step Cloning Kit (Vazyme, Cat# C115). The plasmids were transfected into GC cells using Hieff Trans™ Liposomal Transfection Reagent (Yeasen, Shanghai, China) according to the manufacturer’s instructions.

### *H. pylori* culture and infection assay

*H. pylori* culture and infection assay were performed as previously described [59]. The standard *H. pylori* strain 26695, kindly provided by Dr. Jianzhong Zhang (Chinese Disease Control and Prevention Center, Beijing, China), was inoculated into Brucella broth containing 5% FBS under microaerophilic conditions (5% O_2_, 10% CO_2_, and 85% N_2_) at 37 °C. For *H. pylori* infection, the GC cells were seeded in six-well plates and cultured to 80–90% confluence with an antibiotic-free cell culture medium. Then, the *H. pylori* were harvested, washed with PBS, and added to GC cells at a multiplicity of infection (MOI) of 0, 50, 100, and 150 for 8 h or at a MOI of 100 for 2, 4, and 8 h. Then, the infected cells were collected.

### Cell counting kit-8 (CCK-8) assay

Cells were harvested at 24 h after transfection and seeded into 96-well plates at 3000 cells/well. The mixture of 10 μL CCK-8 solution (TargetMol, Cat# TP1197) and 90 μL RPMI- 1640 medium supplemented with 10% FBS was added to each well, after which the cells were cultured for 2 h. Then, the absorbance at 450 nm and 650 nm was recorded.

### EdU assay

EdU assays were performed using a BeyoClick™ EdU cell proliferation assay kit (Beyotime, Cat# C0075) according to the manufacturer’s instructions. Briefly, the transfected cells were harvested at 24 h after transfection and seeded into 96-well plates. Then, the cells were treated with 5-ethynyl-2′-deoxyuridine and incubated at 37 °C for 2 h. Following fixation with 4% paraformaldehyde, the cells were washed three times with PBS and then incubated at room temperature in a permeabilization solution for 15 min. After another wash with PBS, they were incubated in the dark at room temperature for 30 min with freshly prepared Click Reaction Buffer. Finally, the cell nuclei were stained with Hoechst 33342 for 10 min. Images were captured using a Nikon inverted fluorescence microscope. EdU-positive cells were counted.

### Colony formation assay

Treated cells were seeded into 6-well plates (500 cells/well) and cultured in RPMI-1640 medium supplemented with 10% FBS at 37 °C for approximately 12 to 15 d. The cell colonies were fixed in 4% paraformaldehyde, stained with crystal violet (Solarbio, Cat# G1062) and counted. The experiments were performed in triplicate.

### Transwell assay

The 6.5 mm Transwell® with 8.0 µm pore polycarbonate membrane insert (Corning, Cat# 3422) was used to detect the migration ability of GC cells. Approximately 30,000 cells were seeded into the upper chamber with serum-free 1640 medium, whereas 20% FBS 1640 medium was added to the bottom chamber. After being incubated for 48 h, the cells that migrated to the lower side of the membrane were fixed with methanol and stained with crystal violet.

### Western blot

The cultured cells or gastric tissue samples were washed with PBS and then lysed in RIPA buffer (Beyotime, Cat# P0013B) containing protease inhibitor (Solarbio, Cat# 329-98-6) on ice for 30 min, followed by centrifugation at 12,000 × *g* at 4 °C for 15 min to extract cell proteins. The total proteins (20 μg) were then separated by SDS-PAGE and transferred onto PVDF membranes. After blocking with 5% nonfat milk, the membranes were incubated with the corresponding primary antibodies, washed with TBST, incubated with anti-mouse or anti-rabbit secondary antibodies and visualized using the Super ECL Detection Reagent (YEASEN, Cat# 36208ES76). The antibodies used in this study are listed in Supplementary Table S3. Full-length original western blot results in this article are provided in the Related Manuscript File.

### RNA extraction and real-time qPCR analysis

We extracted total RNA from cells using the TRIzol Reagent (Vazyme, Cat# R401-01) according to the manufacturer’s guidelines. For cDNA synthesis, 1 μg of total RNA was reverse transcribed in a 20 μL reaction volume with a HiScript II 1st Strand cDNA Synthesis Kit (Vazyme, Cat# R212-02). cDNAs were amplified by RT-qPCR using 2× SYBR Green qPCR Mix (SparkJade, Cat# AH101). The relative expression of the target genes was standardized to that of the GAPDH or β-actin and calculated based on the 2^-ΔΔCt^ method. The qPCR primers used are shown in Supplementary Table S4. The validation of the amplification efficiency of the qPCR primers and original Ct value were provided in the Related Manuscript File.

### RNA stability assay

Transfected cells were seeded in six-well plates, treated with Act D (MCE, Cat# HY-17559) at a final concentration of 5 μg/mL, and then collected at the indicated time points. The total RNA was extracted with TRIzol Reagent using the method described above. The relative expression of target genes was detected with RT-qPCR.

### MeRIP-seq

MeRIP-seq was performed by Cloudseq Biotech, Inc. (Shanghai, China). The library was constructed using a NEBNext® Ultra II Directional RNA Library Prep Kit (NEB). The quality of the library was evaluated with a BioAnalyzer 2100 system (Agilent). Library sequencing was performed on an Illumina HiSeq instrument with 150 bp paired-end reads. We used the Integrative Genomics Viewer for m^6^A peak visualization.

### RNA immunoprecipitation (RIP) and MeRIP-qPCR

RIP experiments were performed by using an RNA-binding protein immunoprecipitation kit (BerSinBio, Cat# Bes5101) according to the manufacturer’s instructions. MeRIP experiments were conducted with a m^6^A antibody and an N^6^-Methylated RNA Immunoprecipitation (MeRIP) Kit (BersinBio, Cat# Bes5203) following the manufacturer’s instructions. The immunoprecipitated RNA and corresponding input samples were extracted with a total RNA isolation kit and then subjected to RT-qPCR detection.

### RNA pull-down assay

Biotinylated STAT5A RNA was generated by using a 10 × Biotin RNA labeling mix (Roche, Cat# 1165597910) and a Transcript Aid T7 High Yield Transcription Kit (Thermo Fisher Scientific, Cat# K0441) according to the manufacturer’s instructions. A total of 1 × 10^7^ MKN-45 cells were collected and lysed in 1.7 ml lysis buffer. The supernatant was collected, and 100 µl of the supernatant was reserved for use as an input. The remaining supernatant was then divided into two parts and incubated separately with control probes or specific probes at 4 °C overnight using an RNA pull-down kit (BersinBio, Cat# Bes5102). The pulled-down protein samples were then analyzed by western blot.

### ChIP assay

The ChIP assay was performed using a SimpleChIP® Enzymatic Chromatin IP Kit (Agarose Beads) (CST, Cat# 9002). 2 × 10^7^ cells in each group were treated with 1% formaldehyde and incubated for 10 min before adding glycine. Following the manufacturer’s protocol, the crosslinked chromatin was fragmented and incubated overnight with a Flag antibody (Sigma, Cat# F1804) or negative control IgG (Abmart, Cat# B30011) at 4 °C. After being eluted and purified, the DNA sample was subjected to qPCR detection.

### Dual-luciferase reporter assay

The cDNA fragment of the STAT5A 3’UTR was inserted between luciferase and Renilla in the pmirGLO vector. The constructed pmirGLO-STAT5A-3’UTR vector was transfected into GC cells, together with or without the METTL3 expression vector. The DNA fragment of the STAT5A promoter or the KLF4 promoter was inserted into the upstream of the luciferase reporter gene of the pGL3-basic vector. The STAT5A promoter luciferase reporter vector and pRL-TK vector were cotransfected with the METTL3 expression vector or the control vector. The KLF4 promoter luciferase reporter vector and pRL-TK vector were cotransfected with the STAT5A expression vector or the control vector. After 48 h, the transfected cells were lysed with Passive Lysis Buffer and then subjected to luciferase activity detection according to the instructions of the Promega Dual-Glo Luciferase Assay system.

### Animal experiments

BGC-823 cells were infected with lentivirus harboring the STAT5A shRNA vector or control vector and selected with 2 μg/mL puromycin to obtain a stable cell line. For the subcutaneous xenografts experiment, ten five-week-old male BALB/c nude mice were randomly injected with 5 × 10^5^ BGC-823 control or BGC-823-shSTAT5A cells following the principles of “complete randomization”. Tumor size was monitored by measuring the length (L) and width (W) of the tumor every 2 d with a caliper, and the tumor volume (V) was calculated with the formula *V* = 1/2 × *L* × *W*^2^. Three weeks after injection, the mice were euthanized, and the tumors were weighed. For the nude mouse tail vein injection experiment, 6 × 10^5^ BGC-823 control or BGC-823-shSTAT5A cells were injected into the tail vein of nude mice following the principles of “complete randomization”. The nude mice were weighed every 2 days beginning on the 44th day. On the 55th day, the mice were euthanized. The metastatic nodules in the lungs were counted. The lungs of the nude mice were collected, weighed, and subjected to hematoxylin-eosin staining. The mouse experiments were approved by the ethics committee of the School of Basic Medical Sciences, Shandong University, and were performed with the guidance of animal experiments in the Laboratory Animal Center of Shandong University.

### Human GC samples

As previously reported [60], 35 paired human GC and adjacent noncancerous tissues were collected from Tai’ An City Central Hospital in 2016-2018. All the experiments were approved by the ethics committee of the School of Basic Medical Sciences, Shandong University.

### Statistical analysis

Statistical analysis was conducted using GraphPad Prism version 9.5.0 software. Statistical significance was calculated using Student’s *t*-test (two-tailed) or two-way ANOVA. Correlation analysis was performed using Pearson’s correlation coefficients. Each experiment was performed at least 3 times, and quantitative data are shown as the mean ±S.D. *P < 0.05 **P < 0.01; ***P < 0.001; ****P < 0.0001.

### Supplementary information

Supplementary information

## Data Availability

All data generated or analyzed during this study are available from the corresponding author upon reasonable request.
