# Supplementary material for: METTL3-mediated N6-methyladenosine modification of STAT5A promotes gastric cancer progression by regulating KLF4
Source: Oncogene. 2024 Jun 15;43(30):2338–54. doi: 10.1038/s41388-024-03085-2 (PMC11271408; doi:10.1038/s41388-024-03085-2)
Supplement: Supplementary file 1 — Supplementary information [file 41388_2024_3085_MOESM1_ESM.docx]

**Supplemental information**


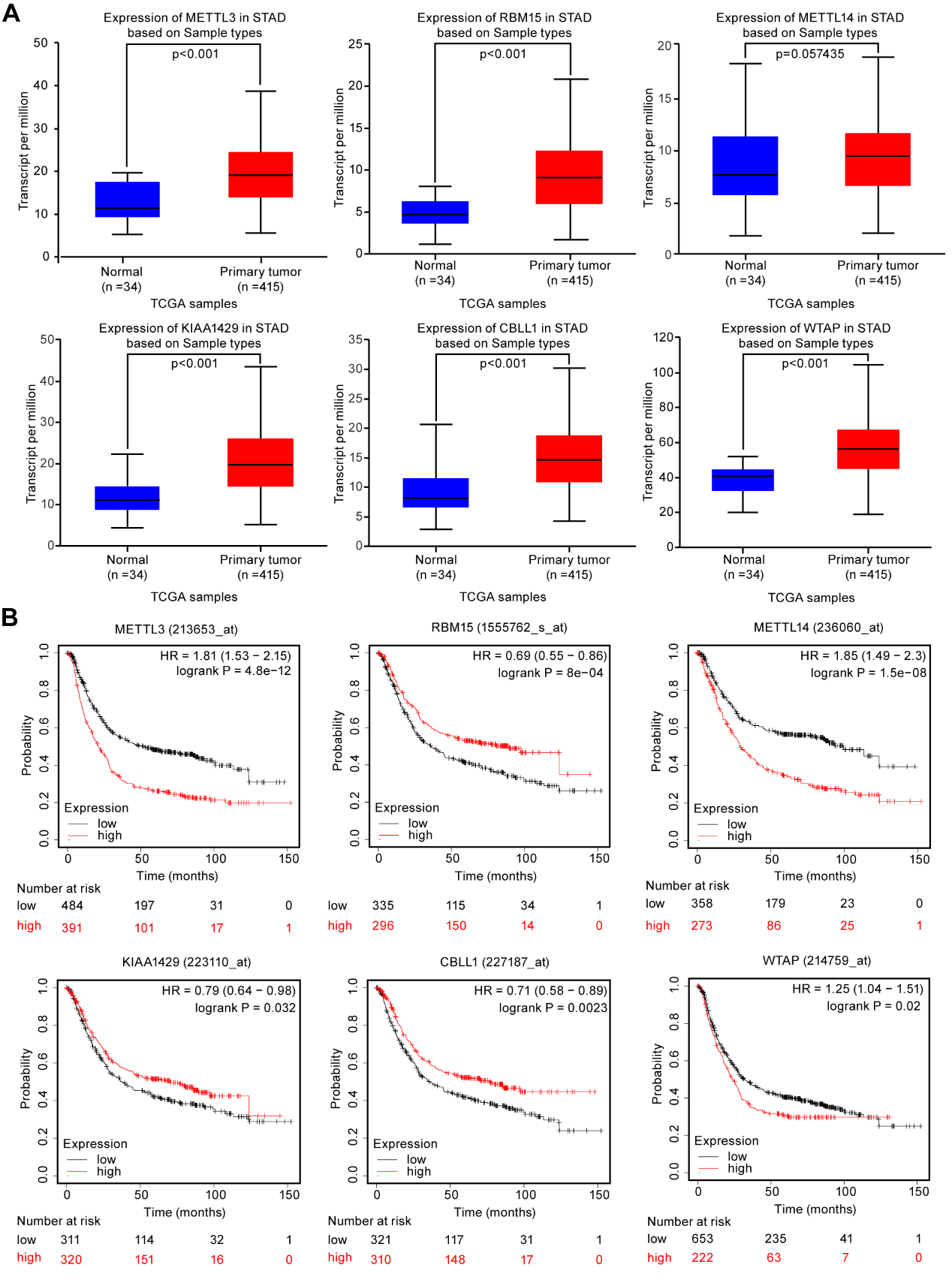


**Figure S1. Expression and prognosis analysis of m^6^A methyltransferase complex members in GC tissues**

**A.**  UALCAN platform was utilized to analyze the mRNA expression levels of METTL3, RBM15, METTL14, KIAA1429, CBLL1 and WTAP in GC tissues (n = 415) and non-tumor tissues (n = 34) based on the data in TCGA database. **B.** Correlation analysis between the expression of METTL3, RBM15, METTL14, KIAA1429, CBLL1 and WTAP and the overall survival (OS) of patients with GC (n = 875) using the data in Kaplan–Meier Plotter database.

**
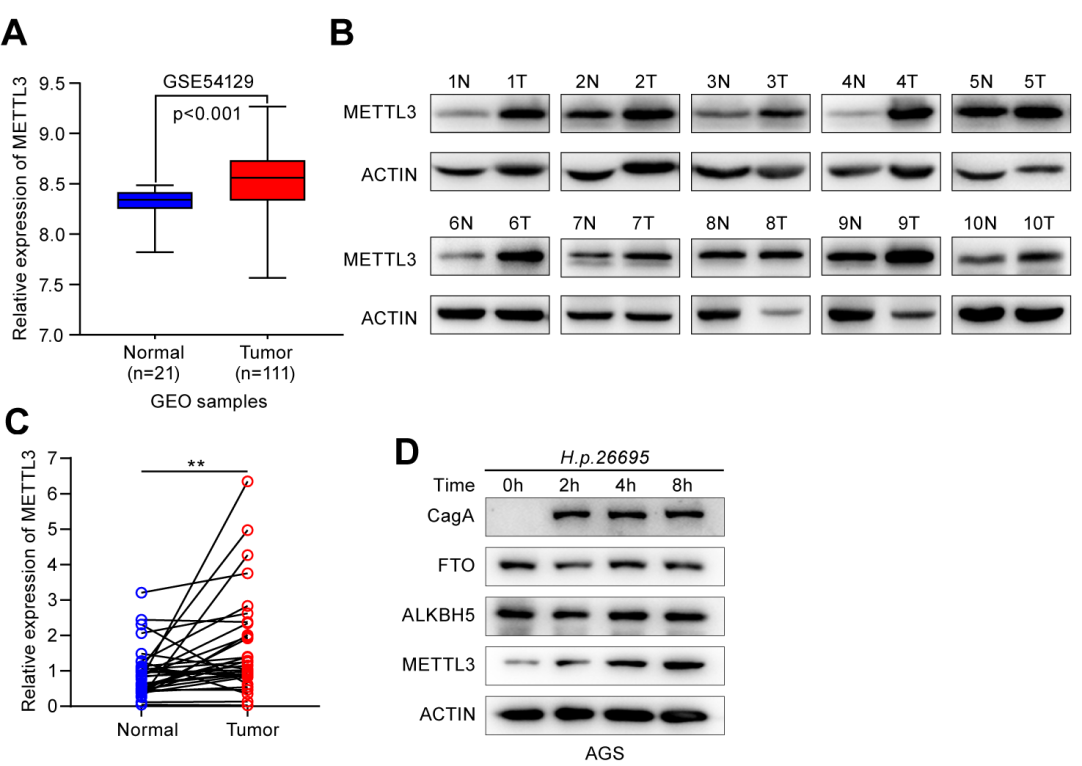
**

**Figure S2. METTL3 is highly expressed in GC tissues**

**A.** Analysis of METTL3 mRNA expression in GC tissues (n = 111) and non-tumor tissues (n = 21) using the data in the GEO (GSE54129) database. **B.** Representative Western blot results to show METTL3 expression in GC tissues and corresponding adjacent noncancerous tissues. N: noncancerous tissue; T: tumor tissue. ACTIN was used as the internal control. **C.** The protein bands of METTL3 and ACTIN were quantified with ImageJ software. Statistical analysis of relative METTL3 protein expression in GC tissues and adjacent noncancerous tissues (n=35) was performed using paired Student's *t* tests. **D.** The protein expression of FTO, ALKBH5, and METTL3 in AGS cells following infection with the *H.pylori 26695* strain for 2, 4, or 8 h at a MOI of 100:1 was evaluated by Western blot. The virulence factor CagA was examined to validate the infection of *H. pylori* in AGS cells. ***P*<0.01.


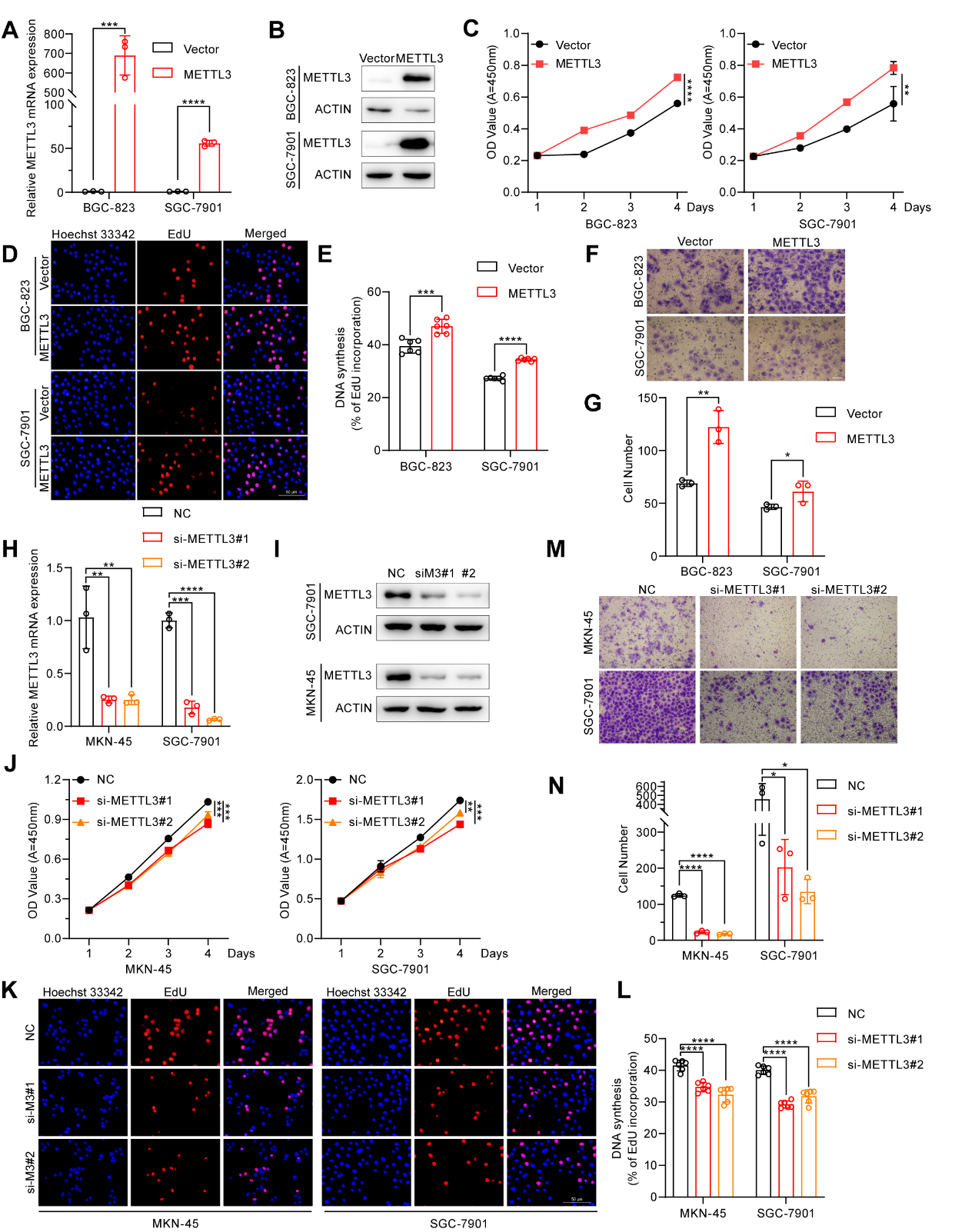


**Figure S3. METTL3 increases GC cell proliferation and migration in vitro**

**A, B.** METTL3 mRNA and protein expression levels were determined by RT‒qPCR (A) and Western blot (B) in GC cells transfected with empty vector (vector) or METTL3 expression vector (METTL3). **C-E.** The effect of METTL3 overexpression on GC cell proliferation was assessed by CCK-8 assay (C) and EdU assay (D, E). **F, G.** Transwell assay was used to detect the migration ability in GC cells transfected with empty vector or METTL3 expression vector. Representative images (F) and statistical analysis (G) were shown. **H, I.** RT‒qPCR (H) and western blot (I) were performed to assess the knockdown efficiency of the two siRNAs against METTL3. siM3: METTL3 siRNA. **J, K, L.** The effect of STAT5A knockdown on GC cell proliferation was assessed by CCK-8 (J) and EdU assay (K, L). si-M3: METTL3 siRNA. **M, N.** Transwell assays were used to detect the effect of METTL3 knockdown on the migration of GC cells. The data are presented as the means ± SD from three independent experiments. **P*<0.05; ***P*<0.01; ****P*<0.001; *****P*<0.0001.


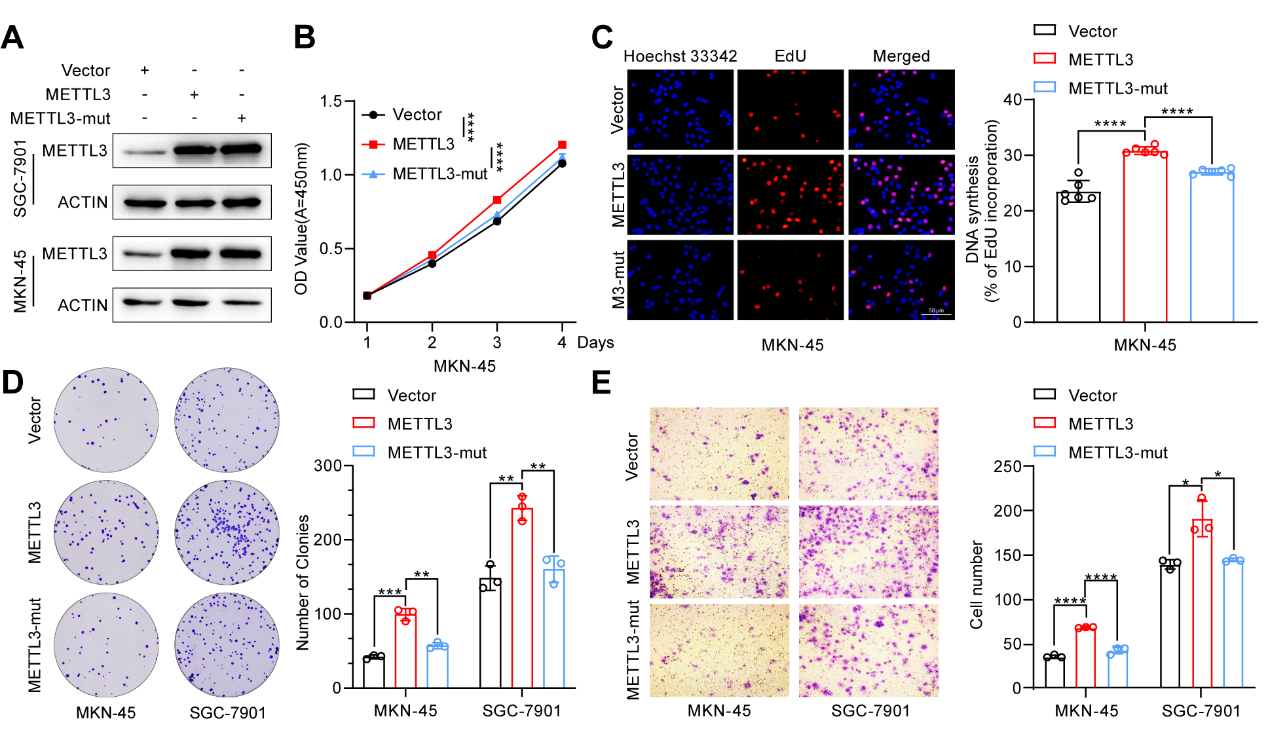


**Figure S4. METTL3 facilitates GC cell proliferation and migration depending on its RNA binding ability**

**A.** METTL3 expression was detected by Western blot in SGC-7901 and BGC-823 cells transfected with empty vector (vector), wild-type METTL3 (METTL3) or mutated METTL3 (METTL3-mut) expression vector. **B, C, D.** Cellular proliferation ability was determined by CCK-8 assay (B), EdU assay (C), and colony formation assay (D) in GC cells transfected with empty vector, wild-type METTL3 or mutated METTL3 expression vector (n = 3). M3-mut in (C): mutated METTL3 expression vector. **E.** Cell migration ability was determined by Transwell assay in MKN-45 and SGC-7901 cells transfected with empty vector, wild-type METTL3 or mutated METTL3 expression vector. Left panel: Representative image; Right panel: Statistical analysis of the number of cells that passed through the Transwell chamber. The data are presented as the means ± SD from three independent experiments. **P*<0.05; ***P*<0.01; ****P*<0.001; *****P*<0.0001.


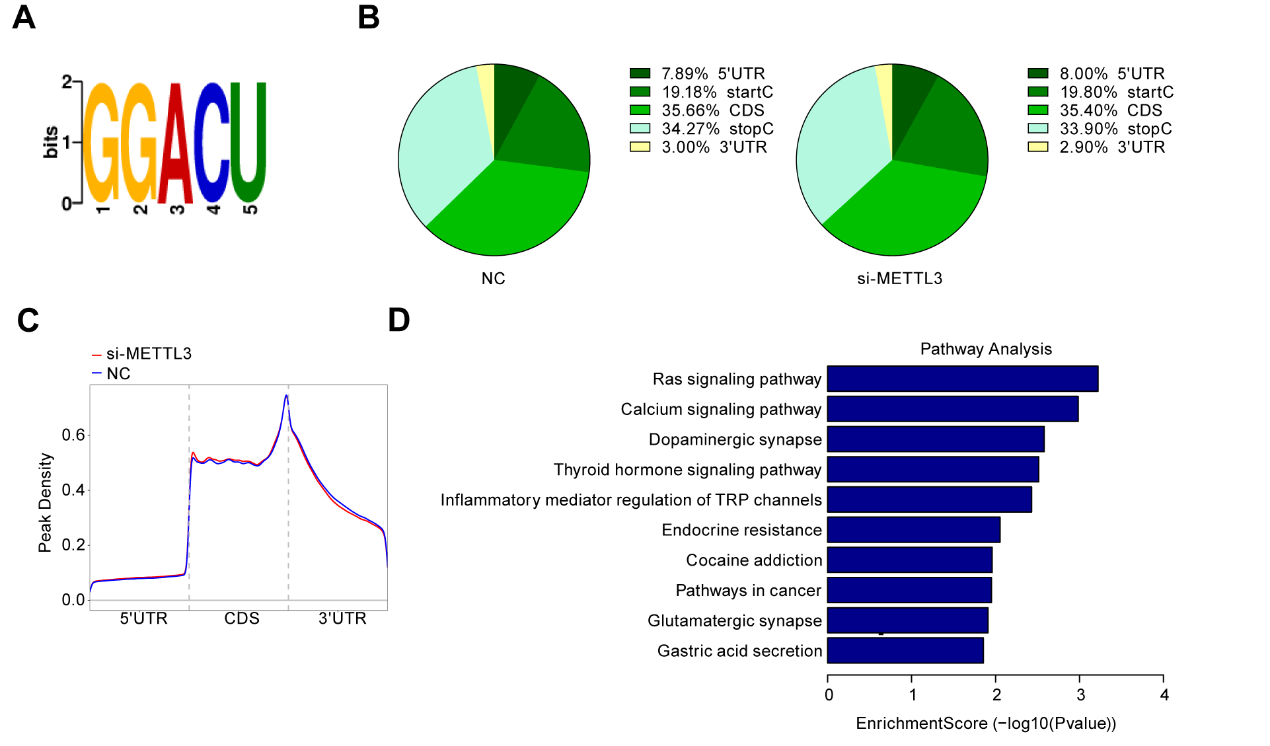


**Figure S5. m^6^A-Seq analysis of the differentially enriched mRNA in GC cells transfected with control siRNA or METTL3 siRNA**

**A.** GGACU motif was identified as the predominant consensus motif in both control and METTL3-deficient cells. **B, C.** The proportions (B) and peak density (C) of the distribution of m^6^A peaks across the entire set of mRNA transcripts were analyzed in terms of their proportions in the 5’UTR, start codon region, CDS, stop codon region, and 3’ UTR. **D**. KEGG pathway enrichment analysis of differentially methylated genes.


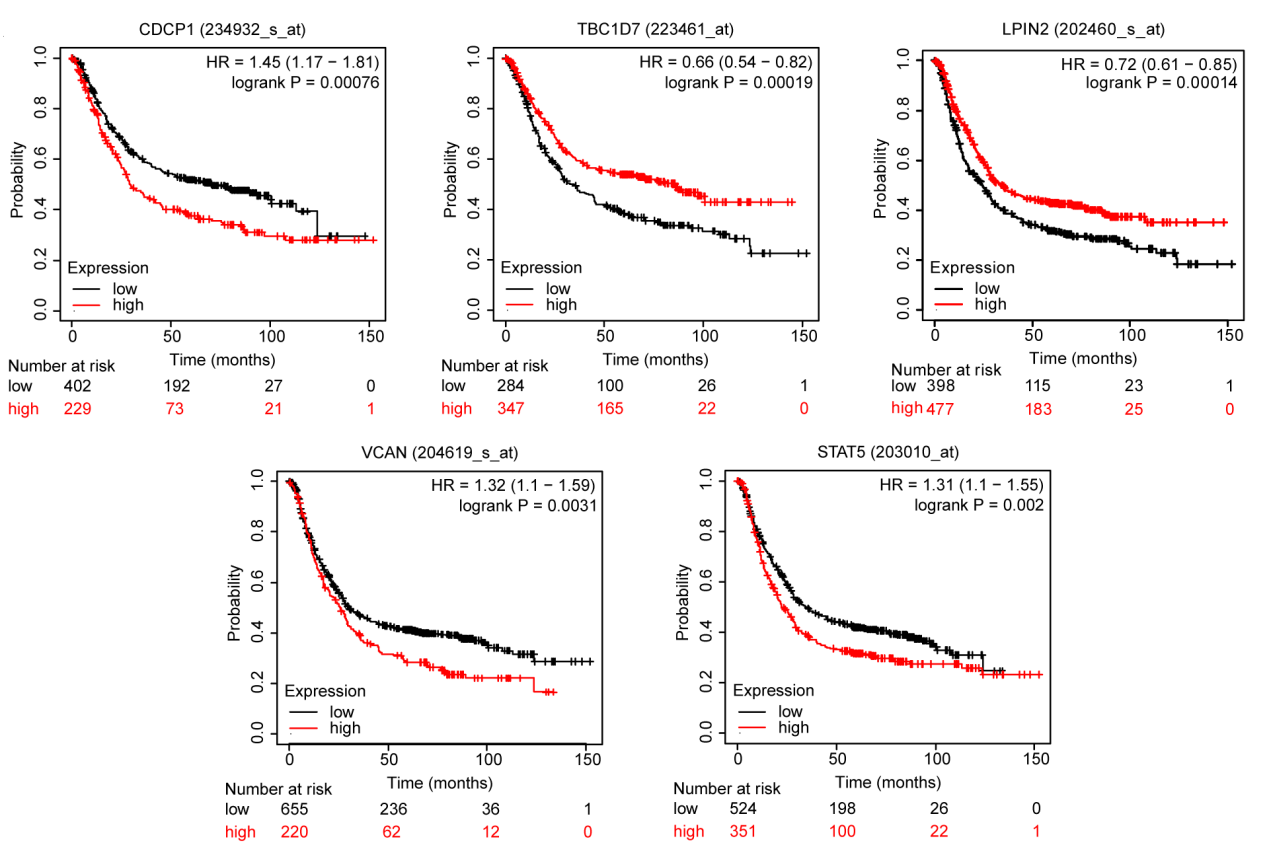


**Figure S6. Correlation analysis between the expression of CDCP1, TBC1D7, LPIN2, VCAN, and STAT5A and the overall survival (OS) of patients with GC (n = 875) using the Kaplan-Meier Plotter database**


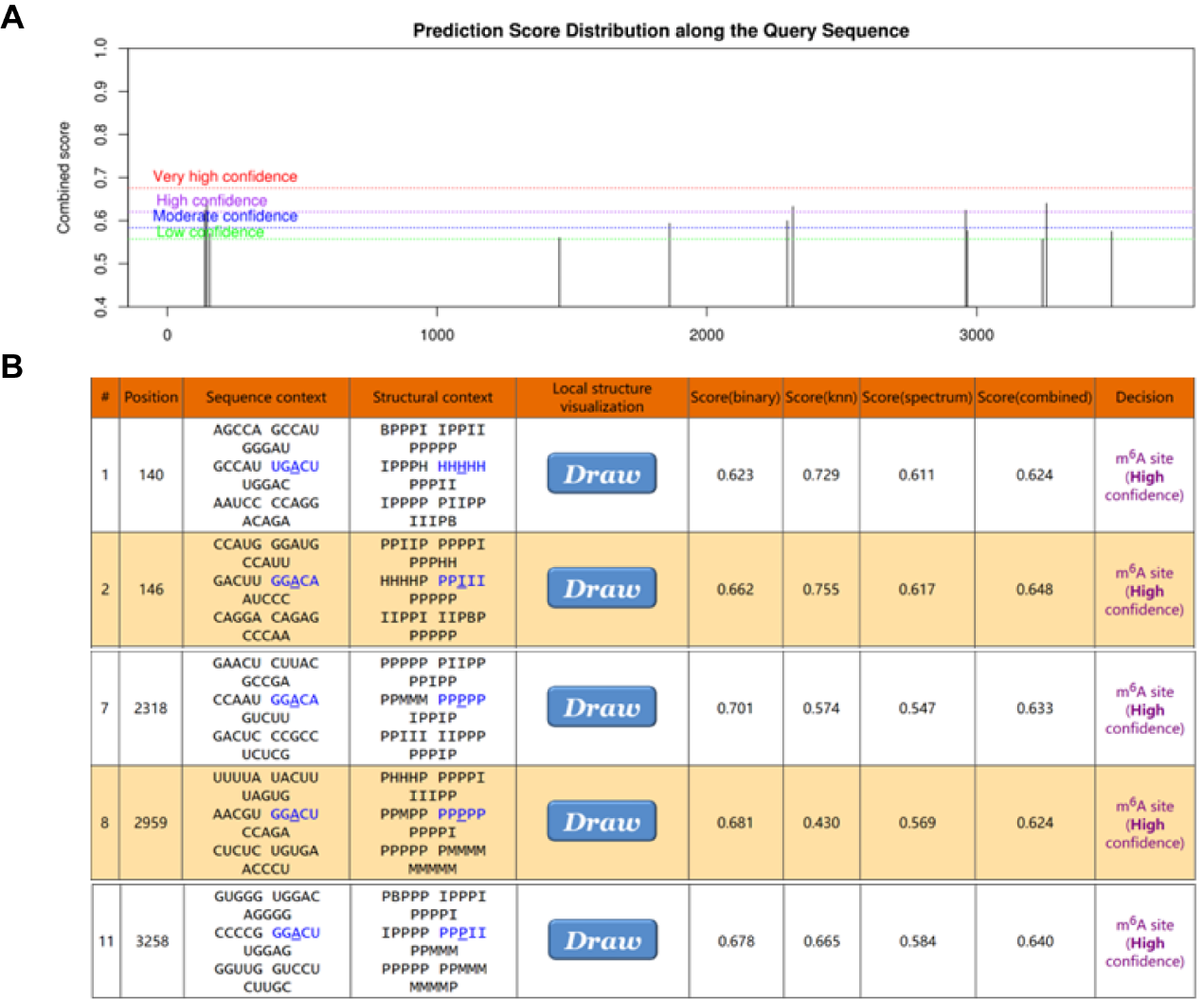


**Figure S7. The predicted results of STAT5A m^6^A modification sites**

**A.** The m^6^A modification site of STAT5A was predicted using the SRAMP database (http://www.cuilab.cn/sramp). **B.** Sequence information of five sites with high confidence.


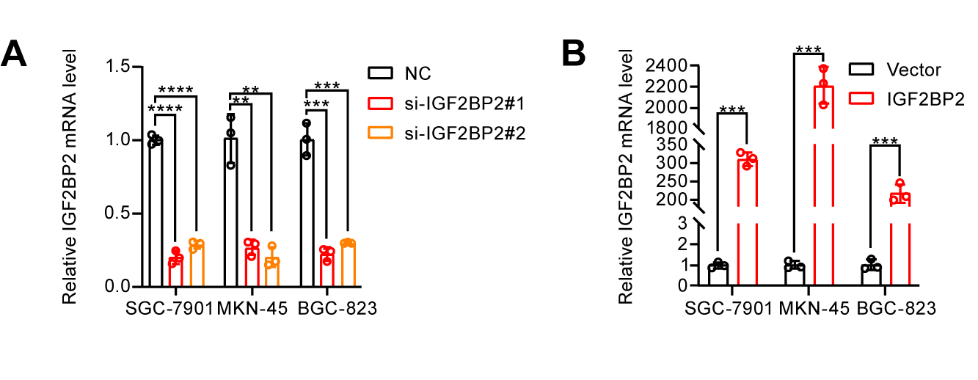


**Figure S8. Validation of transfection efficiency of IGF2BP2**

**A.** RT-qPCR was used to determine the relative mRNA expression of IGF2BP2 in SGC-7901, MKN-45, and BGC-823 cells transfected with negative control siRNA (NC) or METTL3 siRNA (si-IGF2BP2#1/ #2). **B.** RT-qPCR was used to determine the relative mRNA expression of IGF2BP2 in SGC-7901, MKN-45, and BGC-823 cells transfected with empty vector (Vector) or IGF2BP2 overexpression vectors (IGF2BP2). ***P*<0.01; ****P*<0.001; *****P*<0.0001


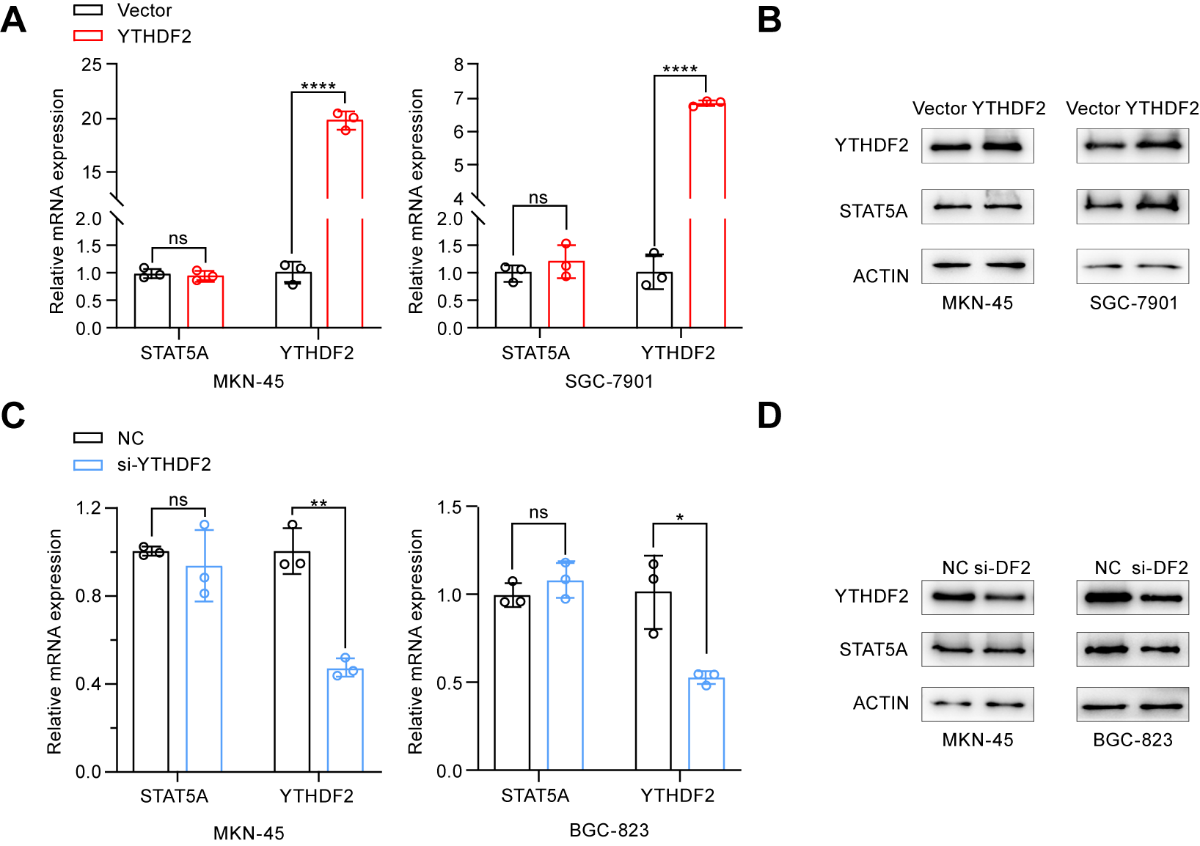


**Figure S9. YTHDF2 had no regulatory effect on STAT5A expression in GC cells**

**A, B.** RT‒qPCR (A) and Western blot (B) were used to determine the relative mRNA and protein expression of STAT5A and YTHDF2 in MKN-45 and SGC-7901 cells transfected with an empty vector or YTHDF2 expression vector. **C, D.** RT‒qPCR (C) and Western blot (D) were used to determine the relative mRNA and protein expression of STAT5A and YTHDF2 in MKN-45 and BGC-823 cells transfected with negative control siRNA (NC) or YTHDF2 siRNA. si-DF2: YTHDF2 siRNA. The data are presented as the means ± SD from three independent experiments. ns: no significance; **P*<0.05; ***P*<0.01; *****P*<0.0001


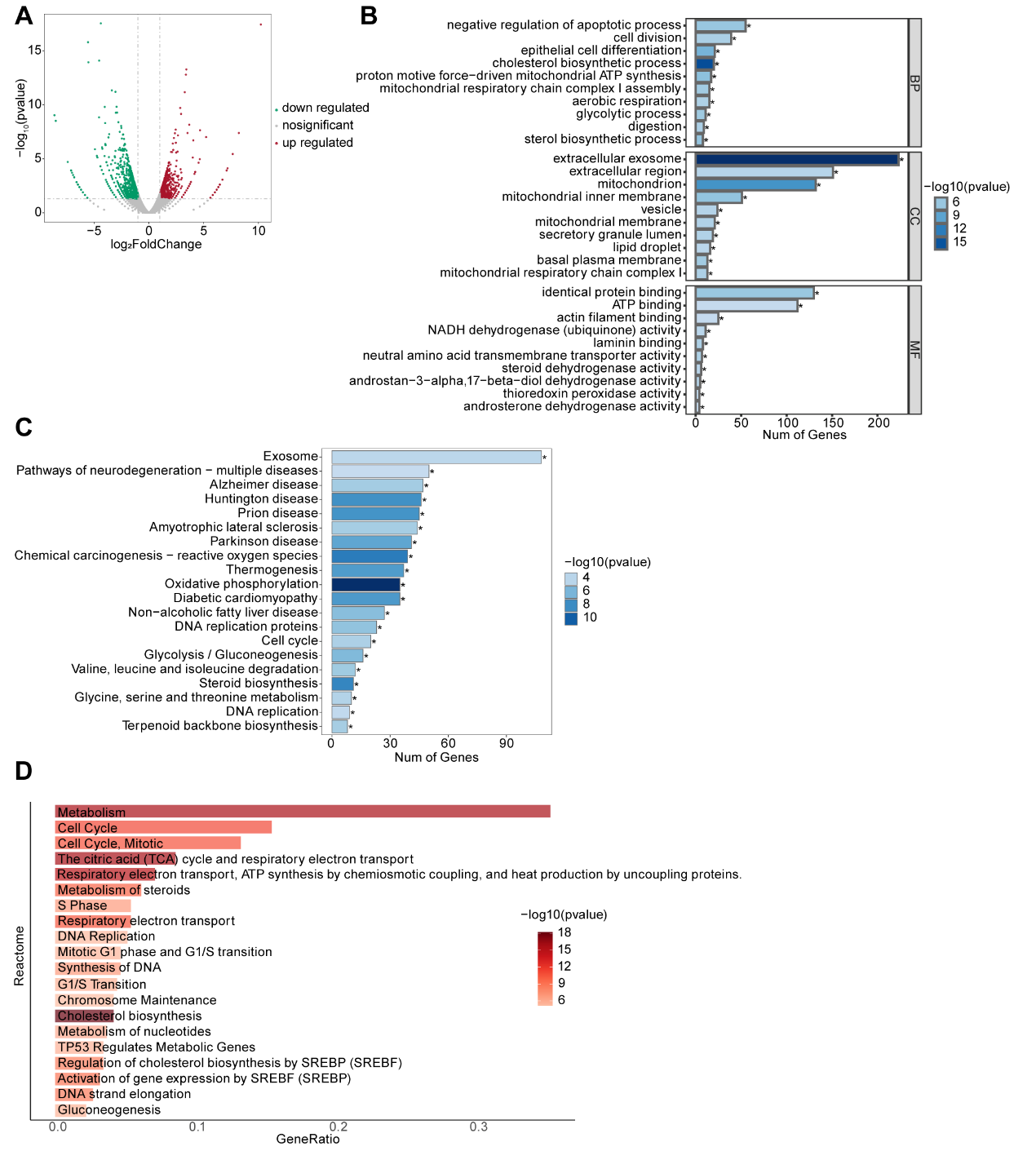


**Figure S10. RNA-seq analysis of AGS cells transfected with empty vector or STAT5A vector**

**A.** Volcano plots of differentially expressed genes in AGS cells transfected with empty vector or STAT5A expression vector. **B-D.** GO (B), KEGG (C), and Reactome (D) pathway enrichment analysis of the differentially expressed genes in RNA-Seq data.


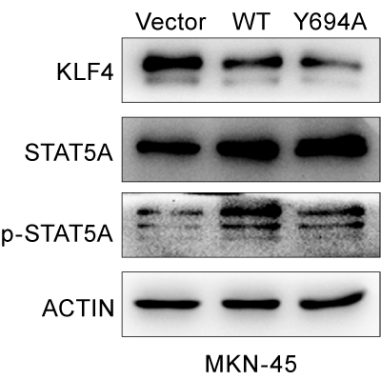


**Figure S11. Western blot analysis of the expression of KLF4 in MKN-45 cells transfected with empty vector, wild-type STAT5A (WT), or STAT5A phosphorylated site mutant (Y694A).**


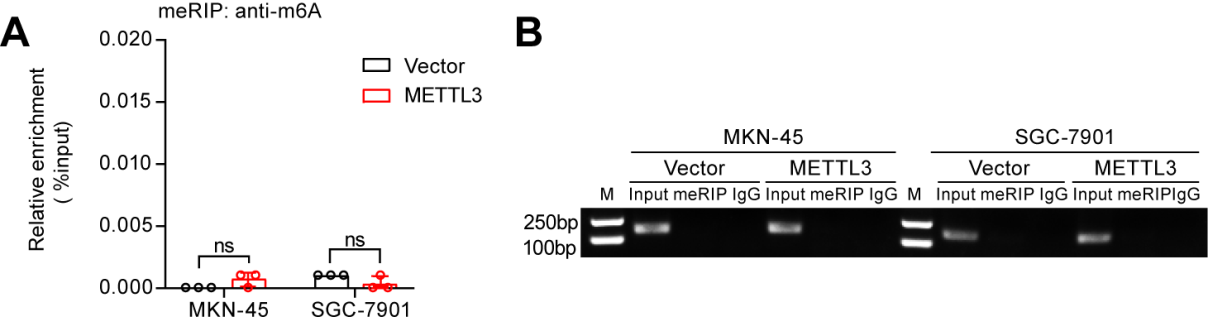


**Figure S12. The m^6^A modification analysis of KLF4 in GC cells**

**A**. MeRIP-qPCR enrichment analysis of KLF4 using m^6^A antibody in METTL3-overexpressing MKN-45 and SGC-7901 cells. **B.** Agarose gel electrophoresis analysis of qPCR products in (A). ns: no significance.

**Supplementary Tables**

**Table S1. siRNA sequences in this study**

| **Name** | **siRNA Sequence** |
| --- | --- |
| METTL3 siRNA1 | 5'-GCUACCUGGACGUCAGUAUTT-3' |
| METTL3 siRNA2 | 5'-GCCAAGGAACAATCCATTGTT-3' |
| IGF2BP1 siRNA | 5'-UGAAUGGCCACCAGUUGGATT-3' |
| IGF2BP3 siRNA | 5'-GCAAAGGAUUCGGAAACTT-3' |
| IGF2BP2 siRNA1 | 5'-ACAGGACUGUCCGUGCUAUTT-3' |
| IGF2BP2 siRNA2 | 5'-GCTGTTAACCAACAAGCCATT-3' |
| STAT5A siRNA1 | 5'-ACAGAACCCUGACCAUGUATT-3' |
| STAT5A siRNA2 | 5'-GUACUACACUCCUGUGCUGTT-3' |
| YTHDF2 siRNA | 5'-GCCCUACUUAACUUCUUAUTT-3‘’ |
| Negative Control | 5’- CCUACAUCCCGAUCGAUGAUGUUGA-3’ |

**Table S2. Oligos sequences for plasmids construction**

| **Name** | **Sequences** | **Note** |
| --- | --- | --- |
| METTL3 mut (sites R295D, K296D,R301D) | F:5’-ACGACCTGCACTTCAGAGATATTATCAATAAACACACTGATGAGTCTTTAG-3’ | Site-Directed Mutagenesis |
|  | R:5’-TCTGAAGTGCAGGTCGTCACAGGGTCGATCAGCATCACTG-3’ |  |
| METTL3 mut  (sites Y331A, F316A,F321A) | F:5’-CCACATGGATACCTGCAAGTATGTTCACGCTGAAATTGATGCTTGCATGGATTC-3’ | Site-Directed Mutagenesis |
|  | R:5’-TTGCAGGTATCCATGTGGGCACATGTATTAAGGGCAGAGCAGTCACCTAAAGACTCATCAG-3’ |  |
| STAT5A-  promoter | F:5’-CATTTCTCTATCGATAGGTACCCTGAACCCCCATCCCGTGGC-3’ | In-Fusion cloning |
|  | R:5’-CAGTACCGGAATGCCAAGCTAGAGCCTCAGTCCCCCTCTCC-3’ |  |
| pGL3 vector for pGL3-STAT5A | F:5’-CTCTAGCTTGGCATTCCGGTACTG-3’ | In-Fusion cloning |
|  | R:5’-GTACCTATCGATAGAGAAATGTTCTGGC-3’ |  |
| STAT5A-  3'UTR | F:5’-TTTAAACGAGCTCGCTAGCCATGTTTGAATCCCACGCTTCTC-3’ | In-Fusion cloning |
|  | R:5’-CAGGTCGACTCTAGACTCGATTTATGAAAAAAATATTTTATTCCAAAACA-3’ |  |
| pmirGLO vector | F:5’-TCGAGTCTAGAGTCGACCTGCA-3’ | In-Fusion cloning |
|  | R:5’-GGCTAGCGAGCTCGTTTAAACA-3’ |  |
| STAT5A-CDS mut1 | F:5’-TGCCATTGGCTTGGACAATCCCCAGGACAGAG-3’ | Site-Directed Mutagenesis |
|  | R:5’-TGTCCAAGCCAATGGCATCCCATGGCTGGCTC-3’ |  |
| STAT5A-CDS mut 2 | F:5’-TTGACTTGGgCAATCCCCAGGACAGAGCCCAA-3’ | Site-Directed Mutagenesis |
|  | R:5’-GGGATTGcCCAAGTCAATGGCATCCCATGGCT-3’ |  |
| STAT5A-CDS mut3 | F:5’-ACCAATGGgCAGTCTTGACTCCCGCCTCTCGC-3’ | Site-Directed Mutagenesis |
|  | R:5’-CAAGACTGcCCATTGGTCGGCGTAAGAGTTCC-3’ |  |
| Truncated IGF2BP2-T1 | F:5’-CAGATTGATCTCGAGGACTACAAAGACCATG-3’ | Truncating Mutagenesis |
|  | R:5’-GTCCTCGAGATCAATCTGTCTGGCCTGAGA-3’ |  |
| Truncated IGF2BP2-T2-1 | F:5’-CACCATGGAAGAGGTGAGCTCCCCTTCGC-3’ | Truncating Mutagenesis |
|  | R:5’-CACCTCTTCCATGGTGGCGGATCCGAGCTCG-3’ |  |
| Truncated IGF2BP2-T2-2 | F:5’-CAGAGCAGCTCGAGGACTACAAAGACCATG-3’ | Truncating Mutagenesis |
|  | R:5’-GTCCTCGAGCTGCTCTGGATAAGAGTGATG-3’ |  |
| Truncated IGF2BP2-T3 | F:5’-CTAGCCTCGAGAGAGTATACAAAAGAAACCATG-3’ | Truncating Mutagenesis |
|  | R:5’-GCTTCTTCATGGTGGCGGATCCGAGCTCG-3’ |  |
| IGF2BP2 KH3-mut | F:5’-CATCGGGGACGACGGGGCACACATCAAACAGCT-3’ | Site-Directed Mutagenesis |
|  | R:5’-GCCCCGTCGTCCCCGATGATGGCGCCCACAGCCTG-3’ |  |
| IGF2BP2 KH4-mut | F:5’-GATTGGCGATGATGGCAAGACCGTGAACGAACT-3’ | Site-Directed Mutagenesis |
|  | R:5’-GCCATCATCGCCAATCACCCGGCCAGCTGT-3’ |  |
| T7-STAT5A-  Sense | F:5’-CGCTAGTAATACGACTCACTATAG-3’ | In-Fusion cloning |
|  | R:5’-TCATGAGAGGGAGCCTCTG-3’ |  |
| T7-STAT5A-  Antisense | F:5’-GAATATAATACGACTCACTATAGGGTCAT-3’ | In-Fusion cloning |
|  | R:5’-ATGGCGGGCTGGATCCAGGC-3’ |  |
| KLF4-promoter | F:5’-CTTACGCGTATTCGGGGCGAGATGCATTCCA-3’ | In-Fusion cloning |
|  | R:5’-GCCAAGCTTACGAAGAGAAGAAACGAAGCCAAAAC-3’ |  |
| pGL3 vector for pGL3-KLF4  promoter | F:5’-CTCTTCGTAAGCTTGGCATTCCGGTACTGTTG-3’ | In-Fusion cloning |
|  | R:5’-GCCCCGAATACGCGTAAGAGCTCGGTAC-3’ |  |
| pGL3-KLF4 MUT Site1 | F:5’-CAAATAAAGACTCCAAACCTAATAGGTACCAGAATCG-3’ | Site-Directed Mutagenesis |
|  | R:5’-GGTTTGGAGTCTTTATTTGTGATACCTGATTTTTCCACTC-3’ |  |
| pGL3-KLF4 MUT Site2 | F:5’-CTGAAAGGATGGTTGACACCAGCCTAAGCGCCAG-3’ | Site-Directed Mutagenesis |
|  | R:5’-GTGTCAACCATCCTTTCAGAAGAGAGGGGACACTGGGGAAAG-3’ |  |
| pGL3-KLF4 MUT Site3 | F:5’-CATTTGGGTCGACCTAAATTACTGATCACAAACCAAGGGG-3’ | Site-Directed Mutagenesis |
|  | R:5’-GTAATTTAGGTCGACCCAAATGTTTTGCTCTCATCTTCTTAGC-3’ |  |
| pGL3-KLF4 MUT Site4 | F:5’-GAGAGTTCTTTCCGAGGGGCGGGGCATGGGAGA-3’ | Site-Directed Mutagenesis |
|  | R:5’-GCCCCTCGGAAAGAACTCTCCGCCCCCCCCGAGGC-3’ |  |
| STAT5A-  Y694A | F:5’-GTTGATGGAGCTGTGAAACCACAGATCAAGCAAGT-3’ | Site-Directed Mutagenesis |
|  | R:5’-GGTTTCACAGCTCCATCAACAGCTTTAGCCAGC-3’ |  |

**Table S3. Antibody informations**

| **Antibodies** | **Source** | **Cat. No** |
| --- | --- | --- |
| ACTIN | Proteintech | 66009-1-Ig |
| METTL3 | Proteintech | 15073-Ap |
| FTO | Abcam | ab126005 |
| ALKBH5 | Abcam | ab195377 |
| CagA | Santa Cruz | sc-28368 |
| STAT5A | Abcam | ab32043 |
| Phospho-STAT5A-Y694 | Abclonal | AP0758 |
| IGF2BP1 | Abcam | ab290736 |
| IGF2BP2 | Proteintech | 11601-1-Ap |
| IGF2BP3 | Abcam | ab177477 |
| FLAG | Sigma | F180F |
| KLF4 | Abcam | ab215036 |
| YTHDF2 | Abcam | ab246514 |

**Table S4. RT-qPCR primer sequences**

| **Name** | **Sequence** |
| --- | --- |
| GAPDH | F:5’-GCACCGTCAAGGCTGAGAAC-3’ |
|  | R:5’-TGGTGAAGACGCCAGTGGA-3’ |
| ACTIN | F:5’-GAAGTGTGACGTGGACATCC-3’ |
|  | R:5’-CCGATCCACACGGAGTACTT-3’ |
| METTL3 | F:5’-ACGGAATCCAGAGGCAGCATTG-3’ |
|  | R:5’-GCGTGGAGATGGCAAGACAGAT-3’ |
| STAT5A | F:5’-AGCAAGTGGTCCCTGAGTTTGTGAA-3’ |
|  | R:5’-CAAGACTGTCCATTGGTCGGCGTAA-3’ |
| IGF2BP2 | F:5’-GATGAACAAGCTTTACATCGGG-3’ |
|  | R:5’-GATTTTCCCATGCAATTCCACT-3’ |
| KLF4 | F:5’-CTACACAAAGAGTTCCCATC-3’ |
|  | R:5’-TGTGTTTACGGTAGTGCCTG-3’ |
| YTHDF2 | F:5’-AGCCCCACTTCCTACCAGATG -3’ |
|  | R:5’-TGAGAACTGTTATTTCCCCATGC -3’ |
| IGF2BP1 | F:5’-TAGTACCAAGAGACCAGACCC-3’ |
|  | R:5’-GATTTCTGCCCGTTGTTGTC-3’ |
| IGF2BP3 | F:5’TATATCGGAAACCTCAGCGAGA-3’ |
|  | F:5’-GGACCGAGTGCTCAACTTCT-3’ |
| VCAN | F:5’-GAAGGCTTGTTTGGACGTTGG-3’ |
|  | R:5’-ACGGAATCCATAAGTCCTGACTC-3’ |
| TNFRSF9 | F:5’-TTGGATGGAAAGTCTGTGCTTG -3’ |
|  | R:5’-AGGAGATGATCTGCGGAGAGT-3’ |
| MXD1 | F:5’-CGTGGAGAGCACGGACTATC-3’ |
|  | R:5’-CCAAGACACGCCTTGTGACT-3’ |
| STMN1 | F:5’-TCAGCCCTCGGTCAAAAGAAT-3’ |
|  | R:5’-TTCTCGTGCTCTCGTTTCTCA-3’ |
| EMP1 | F:5’-GTGTTCCAGCTCTTCACCATGG-3’ |
|  | R:5’-GGAATAGCCGTGGTGATACTGC-3’ |
| CEBPG | F:5’-ACTCCAGGGGTGAACGGAAT-3’ |
|  | R:5’-CATGGGCGAACTCTTTTTGCT-3’ |
| NUDCD1 | F:5’-TTCTCCGTGGAAAGTCAGTGC-3’ |
|  | R:5’-AGCCGTATGGTTACTGTCAAATC-3’ |
| FOS | F:5’-CACTCCAAGCGGAGACAGAC-3’ |
|  | R:5’-AGGTCATCAGGGATCTTGCAG-3’ |
| KLF4 promoter-ChIP-1 | F:5’-CGAGGAGTGGAAAAATCAG-3’ |
|  | R:5’-CCTAGGAAAGAGAAGAGAG-3’ |
| KLF4 promoter-ChIP-2 | F:5’-CGTTTAGAAAGTGATACCGAAC-3’ |
|  | R:5’-GAATAGGAAACGGCGATCTTTAC-3’ |
| KLF4 promoter-ChIP-3 | F:5’-CGCTTGGACGCAGGAGGCG-3’ |
|  | R:5’-CAGAGGCATAAGGGAGAGAAGAGG-3’ |
| KLF4 promoter-ChIP-4 | F:5’-GTGCGCCGAGTTTGTTGATTTAGCTG-3’ |
|  | R:5’-CAGACACGTTCGTTCTCTCTGGTC-3’ |
